# Suicide Inhibition of Cytochrome P450 Enzymes by Cyclopropylamines via a Ring-Opening Mechanism: Proton-Coupled Electron Transfer Makes a Difference

**DOI:** 10.3389/fchem.2017.00003

**Published:** 2017-01-31

**Authors:** Xiaoqian Zhang, Xiao-Xi Li, Yufang Liu, Yong Wang

**Affiliations:** ^1^College of Physics and Materials Science, Henan Normal UniversityXinxiang, China; ^2^State Key Laboratory for Oxo Synthesis and Selective Oxidation, Suzhou Research Institute of LICP, Lanzhou Institute of Chemical Physics, Chinese Academy of SciencesLanzhou, China

**Keywords:** cytochrome P450, hydrogen atom transfer, proton-coupled electron transfer, suicide inhibition, mechanism-based inactivation

## Abstract

*N*-benzyl-*N*-cyclopropylamine (BCA) has been attracting great interests for decades for its partial suicide inactivation role to cytochrome P450 (P450) via a ring-opening mechanism besides acting as a role of normal substrates. Understanding the mechanism of such partial inactivation is vital to the clinical drug design. Thus, density functional theoretical (DFT) calculations were carried out on such P450-catalyzed reactions, not only on the metabolic pathway, but on the ring-opening inactivation one. Our theoretical results demonstrated that, in the metabolic pathway, besides the normal carbinolamine, an unexpected enamine was formed via the dual hydrogen abstraction (DHA) process, in which the competition between rotation of the H-abstracted substrate radical and the rotation of hydroxyl group of the protonated Cpd II moiety plays a significant role in product branch; In the inactivation pathway, the well-noted single electron transfer (SET) mechanism-involved process was invalidated for its high energy barrier, a proton-coupled electron transfer [PCET(ET)] mechanism plays a role. Our results are consistent with other related theoretical works on heteroatom-hydrogen (X-H, X = O, N) activation and revealed new features. The revealed mechanisms will play a positive role in relative drug design.

## Introduction

Suicide inhibitors, or called mechanism-based (*k*_*cat*_) inactivators, are attractive of great interests in enzymology and drug industry, because the action of such inhibitors is intimately related to the enzymatic mechanism, well knowledge of the latter often provides an excellent starting point for the rational design of highly specific and effective drugs in clinical use (*e.g*., penicillin) (Meunier et al., 2004; Ortiz de Montellano and De Voss, 2005). Besides, discovery of such inhibitors for an enzyme, *e.g.*, cytochrome P450, whose mechanisms are not well-characterized should provide an equally specific and effective probe. Metabolism of these alternative substrates could result in the generation of reactive species that inactivates the enzyme through either covalent modification or tight binding (Orr et al., 2012). Cyclopropylamines are one kind of such prototypical inhibitors for cytochrome P450 enzymes (P450s), which are widely found in biologically active natural products, synthetic drugs and also widely used as mechanistic probes to reveal elusive reaction mechanisms, such as that involved in P450-catalyzed amine oxidations (Bhakta and Wimalasena, 2002; Ortiz de Montellano and De Voss, 2002; Totah and Hanzlik, 2002; Bhakta et al., 2005).

**Figure 1:**
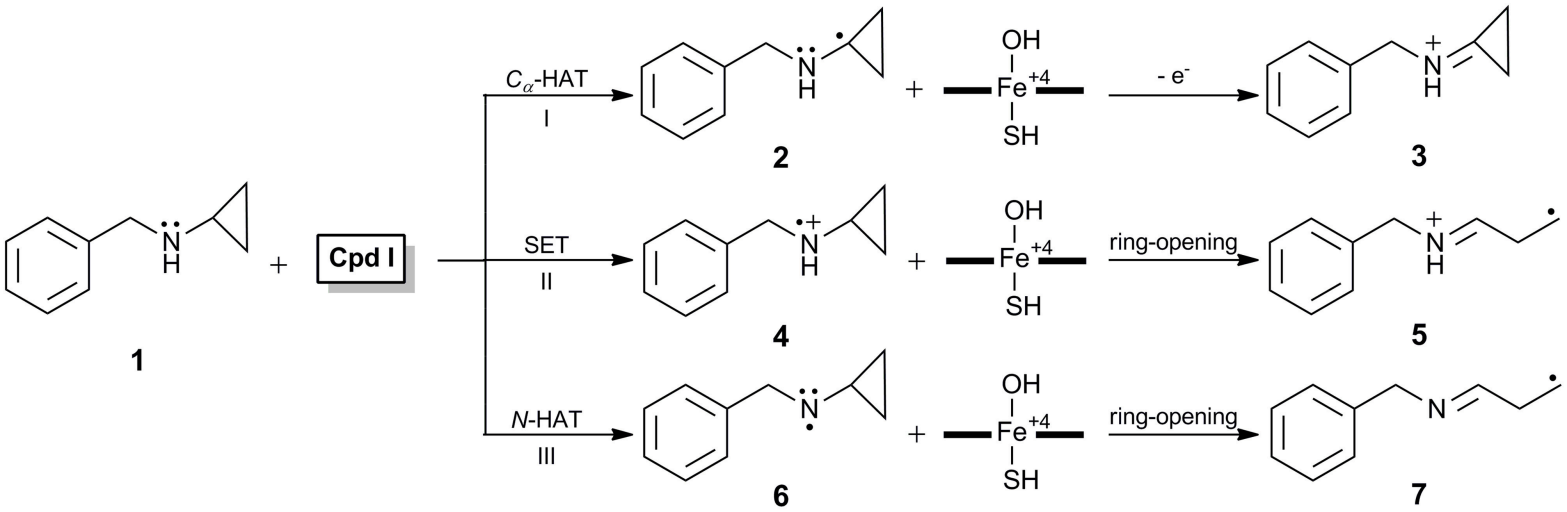
**Proposed mechanisms involved in BCA-induced inactivation of cytochrome P450**.

*N*-benzyl-*N*-cyclopropylamine **1** (Figure 1, hereafter as BCA in brief) functioning as a suicide inhibitor of P450 was first reported by Hanzlik et al. (1979), and toward understand the special inactivation role, they proposed a C_α_-hydrogen atom transfer (C_α_-HAT, **I** in Figure 1) mechanism in which a Schiff base intermediate **3** was deemed to account for the inactivation. However, Hanzlik (Hanzlik and Tullman, 1982) and Guegerich (Macdonald et al., 1982) further found that the 1'-methyl-substituted analog of **1**, *N*-benzyl-*N*-(1′-methylcyclopropyl)amine lacking hydrogen atom at the C_α_ position of cyclopropyl group, was also capable of inactivating P450 almost as effectively as **1**. Thus, the C_α_-HAT mechanism was ruled out. Instead, a single electron transfer (SET) mechanism (Macdonald et al., 1982) (**II** in Figure 1) was postulated that the inactivation involved an initial heteroatom oxidation to aminium cation radical **4**, which could undergo rapid ring-opening process to form highly reactive carbon-centered radical species **5** and subsequently covalently bound to the amino residue within the enzyme active site (Macdonald et al., 1982).

The SET mechanism can also explain some other observations for amine oxidations, such as the inhibition role of 4-alkyl-1,4-dihydropyridine derivatives which could extrude an alkyl radical during the P450-catalyzed oxidation (Augusto et al., 1982), the small deuterated kinetic isotope effect (KIE) (Miwa et al., 1983), the correlation of free energy relationship to one-electron oxidation potential (Guengerich et al., 1984), the large negative Hammett ρ in *N*-demethylations of *para*-substituted *N*,*N*-dimethylanilines by both P450 (Burka et al., 1985) and its nonheme biomimics (Nehru et al., 2007), thusly was widely accepted as a general mechanism involved in amine oxidation (Guengerich et al., 1996). However, the validity of such evidence for the SET mechanism was subsequently challenged. Dinnocenzo et al. found that the KIE profiles for a series of dimethylaniline (DMA) oxidations catalyzed by P450 correlated linearly with those measured in the reactions of the same DMAs with *tert*-butoxyl radical which actually involved a typical hydrogen atom transfer (HAT) mechanism (Dinnocenzo et al., 1993; Karki and Dinnocenzo, 1995; Karki et al., 1995; Manchester et al., 1997). Shaffer et al. reported that the oxidation of *N*-methyl-*N*-cyclopropylaniline by horseradish peroxidase (HRP), a conventional SET oxidant, led to yields of ring-opened intermediates and subsequently inactivated the enzyme, conversely, as an oxidant whose reduction potential was higher, the P450-catalyzed oxidation of *N*-methyl-*N*-cyclopropylaniline produced ring-intact metabolites exclusively and no inactivation was observed (Shaffer et al., 2001a,b). In addition, the use of clock substrate and other experimental criteria seemed to rule out the involvement of the amine cation radical intermediate (Shaik et al., 2010). To determine the complete fate of **1** *in vivo* by P450 as well as to reconcile the discrepant behaviors in HRP- and P450-catalyzed reactions, Cerny and Hanzlik (2005, 2006) performed a series of experiments, and eventually proposed an *N*-HAT mechanism (**III** in Figure 1) through the product study. This novel *N*-HAT mechanism was proposed upon a ring-opening process of the cyclopropyl group, *i.e*., hydrogen abstraction from the N-H bond of the secondary cyclopropylamine **1** gave a neutral aminyl radical species **6** which could undergo rapid ring-opening process to form **7**, such reactive *C*-centered radical species accounted for the enzyme inactivation by means of covalently binding to the amino residue in the active site. Moreover, such *N*-HAT mechanism was subsequently validated by Hirao et al. in their investigation on P450 inactivation by 1,1-dimethylhydrazine (Hirao et al., 2013a). However, previous investigations have revealed that there might be an alternative proton-coupled electron transfer (PCET) mechanism for the polar X-H (X = O, N) bond activation (Mayer et al., 2002; Usharani et al., 2013; Hirao and Chuanprasit, 2015; Li et al., 2015). Thus, an obvious question to be answered is: Ring-opening of BCA is initiated by a direct HAT mechanism or a PCET one?

Furthermore, according to Hanzlik, the incubation of **1** with microsomes indeed caused a time-, concentration- and cofactor-dependent loss of cytochrome P450 activity, which was, however, not vanished completely but instead remained 25–30% of it (Cerny and Hanzlik, 2005). In an effort to reveal the genuine mechanism of P450 inactivation by *N*-benzyl-*N*-cyclopropylamine that involved in the physiological process, and thereby making contributions to pharmacy, we performed a series of density functional theory (DFT) calculations. Two distinct pathways, inactivation and metabolic ones, were examined in which BCA reacted with P450 efficiently. Moreover, we also demonstrated that in the inactivation pathway, the N-H bond activation/ring-opening process was a prior reaction route for the low energy barrier of its rate-limiting step, 0.6/0.4 kcal/mol for the high quartet spin-state (HS)/low doublet spin-state (LS). Essentially, the validity of the PCET mechanism that involved in the H-abstraction at the N-H bond of BCA by P450 was confirmed.

## Theoretical methods

Due to the key role of the computational modeling on the investigation of enzyme reaction (Li et al., 2012; de Visser et al., 2014), we employed an iron-oxo open-shell porphyrin with an axial thiolate ligand to model CpdI [Fe^4+^O^2−^(C_20_N_4_H_12_)^−^(SH)^−^] (Shaik et al., 2005; Wang et al., 2006, 2007a, 2010), and used the *N*-benzyl-*N*-cyclopropylamine as the substrate. All DFT calculations were performed with the Gaussian 03 suite of quantum chemical packages (Frisch et al., 2004). The spin-unrestricted B3LYP (Lee et al., 1988; Becke, 1992a,b, 1993) functional was employed with two basis sets: (a) The LACVP(Fe)/6-31G^*^(H, C, N, O, S) (denoted as LACVP^*^, henceforth B1) for geometry optimizations without symmetry constraint; (b) The LACV3P(Fe)/6-311++G^**^(H, C, N, O, S) (denoted as LACV3P++^**^, henceforth B2) for single point energy (SPE) calculations (Hay and Wadt, 1985). Transition states were ascertained by vibrational frequency analysis to possess a single mode along the reaction path with only one imaginary frequency. Bulk polarity effects of the active site in the protein environment were evaluated with the polarizable continuum model (PCM) using a nonpolar solvent, chlorobenzene (ε = 5.697).

The ^4(2)^**IM**_SET_ species (whose occupation diagram and Mulliken spin density are shown in Supplementary Figure 2 and Supplementary Table 3) involved in the SET process were obtained by shifting one electron from the highest occupied orbital of *N*-benzyl-*N*-cyclopropylamine (σ_CH_) to the lowest vacant orbital of porphyrin moiety (a_2u_) of CpdI on the reactant complex (^4(2)^**RC**) (Li et al., 2006). Any attempt to optimize the structure of ^4(2)^**IM**_SET_ species will result in their collapse to the ground state.

The kinetic isotope effect for the hydrogen abstraction process was determined using Gaussian frequency data based on the semi-classical Eyring equation (Melander and Saunders, 1987), where the KIE is given as.

(1) $${\left(\frac{k_{H}}{k_{D}}\right)}_{s}=\text{ exp }\left[-\frac{\left(G_{H}^{‡}-G_{H}^{R}\right)-\left(G_{D}^{‡}-G_{D}^{R}\right)}{RT}\right]$$

*k* denotes the reaction rate constant, G is the Gibbs free energy of abstraction, R is the gas constant, T is the absolute temperature.

All data were collected in the Supplementary Material document, while we discussed the highest-level results, UB3LYP/B2//B1, in the text. Thus, the SPE B2//B1 value with the zero point energy (ZPE) correction referred to E1, whereas E2 included the bulk polarity effects and ZPE corrections.

## Results and discussion

### The inactivation pathway

In retrospect, Hirao et al. recently investigated the mechanism-based inactivation of cytochrome P450 by 1,1-dimethylhydrazine (UMDH) (Hirao et al., 2013a), they examined four possible reaction pathways including H-abstractions from the primary amine moiety and the methyl moiety, as well as the direct oxidations on two nitrogen atoms. Owing to the lower energy barriers, they concluded that the H-abstraction from the N-H bond of the primary amine moiety was the most favorable pathway, on which our investigation is based.

**Figure 2:**
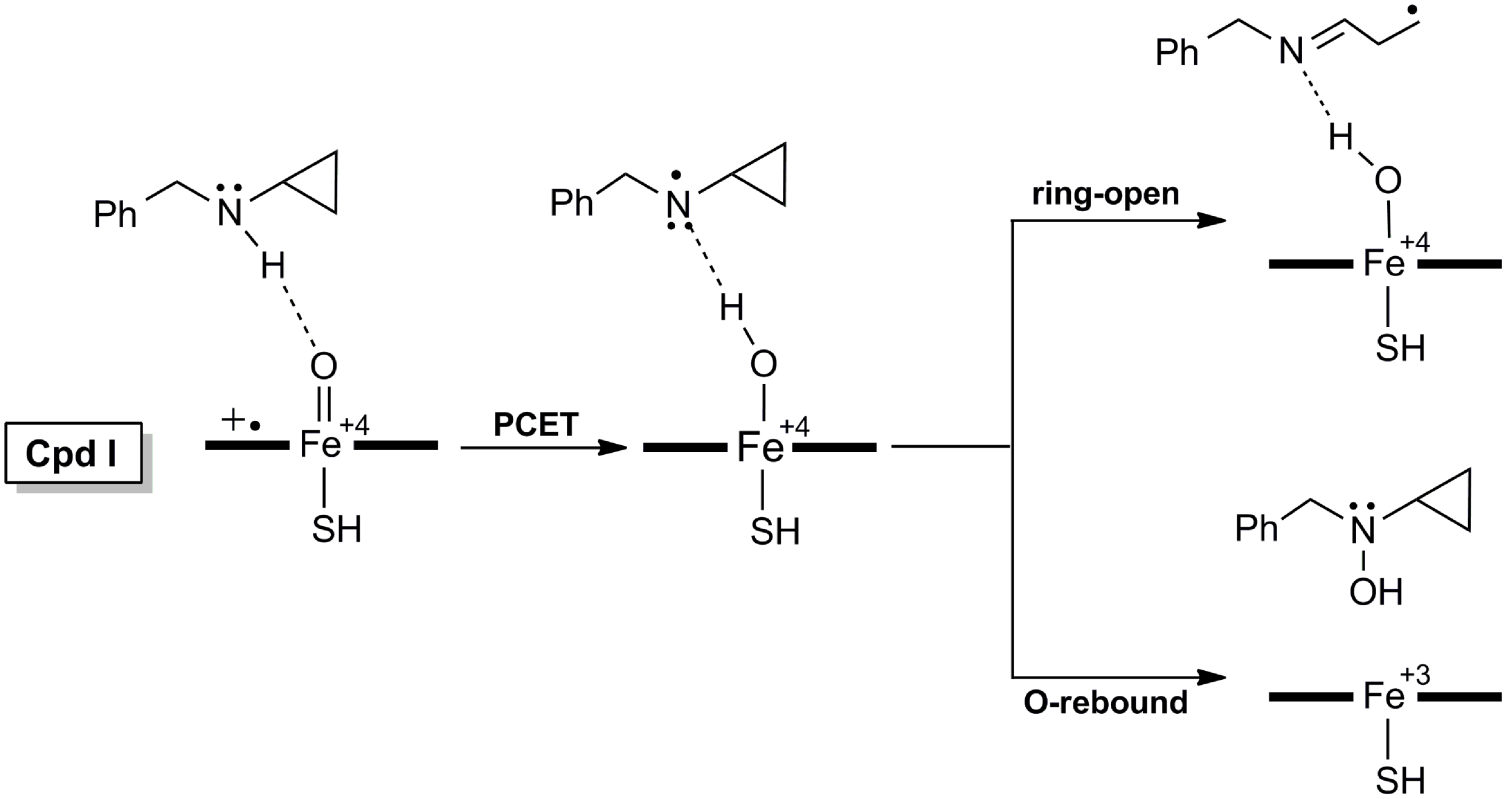
**Proposed mechanisms of H-abstraction and the following ring-opening/O-rebound processes of BCA by P450**.

As for the inactivation of P450 by BCA (Figure 2) which involves the PCET mechanism, the substrate is initially bound to CpdI through the N-H^…^O hydrogen-bonding interaction, the subsequent H-abstraction from the N-H bond results in an amino radical intermediate (**IM** species). Owing to the newly-formed hydrogen-bonding between O-H and N, dichotomous behaviors are encountered at the following step. The amino radical IM species could undergo either a traditional O-rebound process to yield hydroxylamine which has been deemed to be the precursor to oxime (Cerny and Hanzlik, 2005; Hirao et al., 2013b), or a rapid ring-opening of the cyclopropyl group forming a *C*-centered radical species, which is essential for the enzyme inactivation and could be converted to 3HP via a further oxidation.

**Figure 3:**
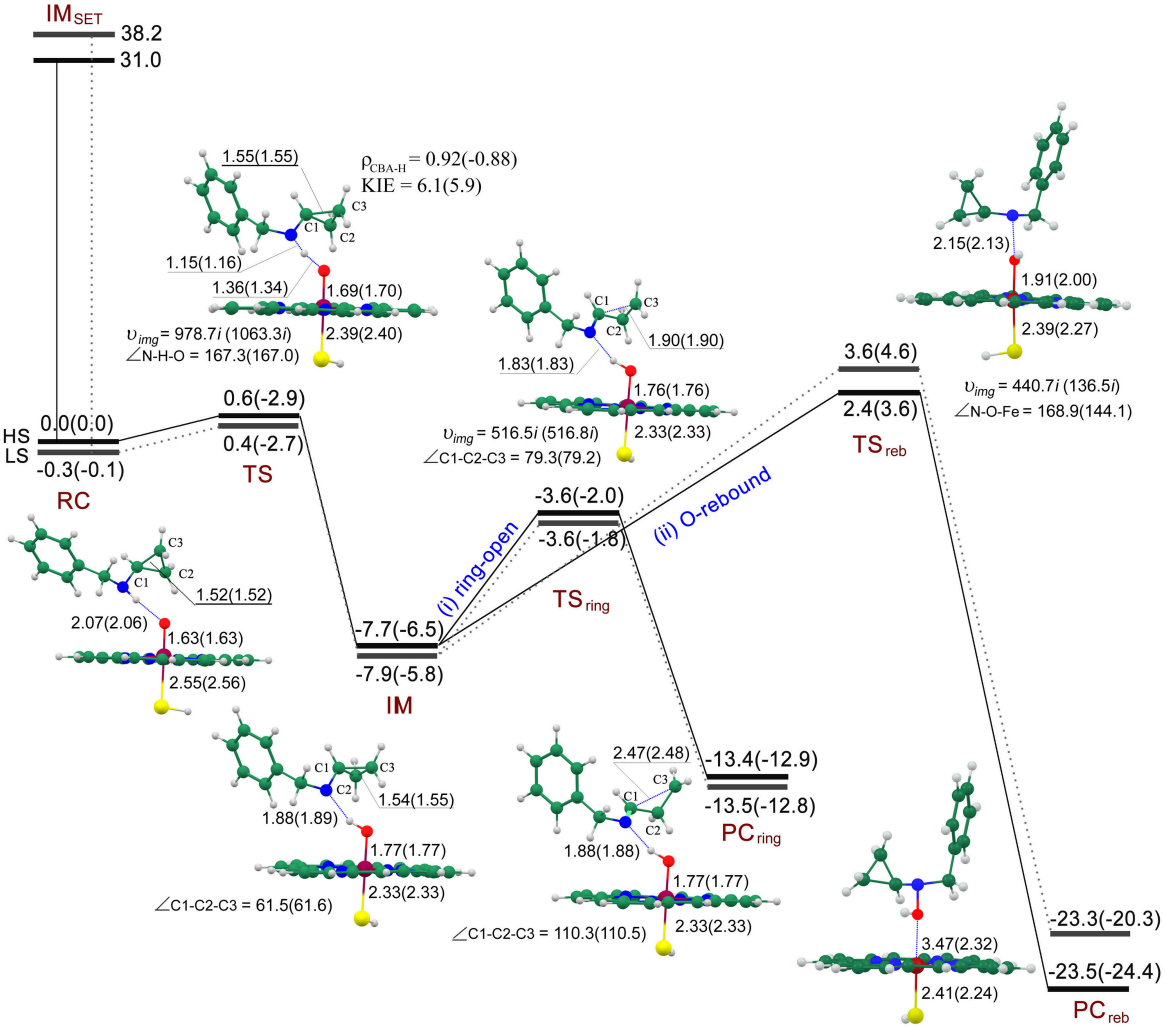
**Energy profiles (UB3LYP/B2//B1) for SET process and the competitive ring-opening/O-rebound step after H-abstraction process at the E1(E2) level and the geometries (UB3LYP/B1) of the key intermediates for the HS(LS) involved**. The relative energy is in kcal/mol, bond length is in Å, bond angle is in degree and the imaginary frequency is in cm^−1^ unit.

On the exploration of the inactivation mechanism of P450 by BCA, two controversial mechanisms (**II** and **III** in Figure 1) have been postulated by Guengerich (Macdonald et al., 1982) and Hanzlik (Cerny and Hanzlik, 2006), respectively. Figure 3 shows the energy profiles for the SET and PCET processes, accompanied by the geometries of the involved key intermediates. First of all, we evaluate the energy barriers of the SET process for both quartet and doublet spin states through DFT calculations. It is obvious that the SET energy barrier (**IM**_SET_, 31.0/38.2 kcal/mol for the HS/LS) is much higher compared with the direct hydrogen abstraction from N-H bond via a PCET mechanism (0.6/0.4 kcal/mol for the HS/LS). Such sharp energetic comparison between SET and PCET processes definitely rules out the former and favors the latter. On **RC**, two spin states are nearly degenerated with the LS lying under the HS by -0.3/-0.1 kcal/mol at the E1/E2 level. Intriguingly, the following essential H-abstraction from the N-H bond of the secondary amine moiety proceeds through an extremely low energy barrier (0.6/0.4 kcal/mol for the HS/LS at the E1 level, indicating an involvement of two-state reactivity mechanism (Shaik et al., 1998; Schröder et al., 2000; de Visser and Tan, 2008), which is similar to our previous finding about the oxidation of 4-alkylated DHPs (Li et al., 2015) as well as that reported by Hirao et al. (2013a) but different from that obtained by Rydberg and Olsen (2011). Hirao et al. attributed the tiny energy barrier to weak bond disassociation energy (BDE) of N-H bond rather than the involvement of PCET mechanism, which has been proved in our previous work. Whereas, in Rydberg's work which studies the P450-catalyzed hydroxylation of propan-2-amine, the energy barrier of such H-abstraction was much higher, 12.6/11.6 kcal for the HS/LS in the gas phase, such significant energetic discrepancy is attributed to that the N-H bond BDE decreases when going from primary to secondary amines (Lalevée et al., 2002; Luo, 2002). The inclusion of the bulk polar effect even changes the H-abstraction into a barrierless process (−2.9/−2.7 kcal/mol for the HS/LS). The **IM** species which comprises the *N*-centered radical substrate and the ferryl-hydroxyl closed-shell porphyrin (protonated CpdII) is generated after the H-abstraction at the N-H bond. Unlike the **IM**_CH_ species in most P450-catalyzed C-H bond hydroxylation which always lies upon the **RC**_CH_ species (Ogliaro et al., 2000; de Visser et al., 2002, 2004; Kumar et al., 2003; Li et al., 2006; Olsen et al., 2006; Wang et al., 2006, 2007a,b, 2010), the **IM** species in N-H bond activation lies under the **RC** species by −7.7/−7.9 kcal/mol for the HS/LS at the E1 level. That is because compared with the **IM**_CH_ in which the carbon radical interacts with the protonated CpdII, the OH^…^N hydrogen bonding in **IM** is quite significant for stabilizing the amino radical, and consequently lowering the systematic energy (Korzekwa et al., 1990; Jones et al., 2002; Olsen et al., 2006). As mentioned above, the following step is dichotomous. Through the novel ring-opening route, the *N*-centered radical rapidly rearranges to a ring-opened *C*-centered radical that could covalently bind to the amino residue within the active site, and therefore inactivating the enzyme. The **TS**_ring_ species is more stable than the **RC** species in an energy of −3.6/−3.6 kcal/mol at the E1 level and −2.0/−1.8 kcal/mol at the E2 level for the HS/LS, while lies 4.1/4.3 kcal/mol higher relative to the **IM** for the HS/LS at the E1 level. Thus, the ring-opening process should be the rate-limiting step on such hydrogen abstraction/ring-opening route. Additionally, the relative “high” energy of the ring-opened product **PC**_ring_ (−13.4/−13.5 kcal/mol for the HS/LS at the E1 level) implies that it may be reactive and could be further oxidized to 3HP. Alternatively on the conventional O-rebound route, it would form hydroxylamine in a same manner as the well-known C-H bond hydroxylation by CpdI (Ogliaro et al., 2000; de Visser et al., 2002; Kumar et al., 2003; Wang et al., 2006). During the O-rebound process, the spin state order exchanges with an energy barrier of 2.4/3.6 kcal/mol for the HS/LS at the E1 level. Such energy barrier is higher than that of the PCET process, thus making the O-rebound process the rate-limiting step on this hydrogen abstraction/O-rebound route. While in P450-catalyzed C-H bond hydroxylations (Ogliaro et al., 2000; de Visser et al., 2002; Kumar et al., 2003; Wang et al., 2006), the O-rebound process proceeds via a quite low energy barrier, even it often becomes barrierless on the doublet spin state. Rydberg et al. also reported the similar higher rebound energy barrier in their recent investigation regarding the P450-catalyzed hydroxylation of primary amines, such distinct energetic discrepancy between C-H bond and N-H bond hydroxylations was ascribed to different radical orientations and the existence of strong O-H^…^N hydrogen-bonding interaction in the intermediate species (Rydberg and Olsen, 2011). On the whole, the energy barrier of O-rebound process is significantly higher than that of ring-opening process by 6–7 kcal/mol, indicating that the hydrogen abstraction/ring-opening route is prior to the hydrogen abstraction/O-rebound one. Such conclusion also coincides with the observation of the major ring-opened product, *i.e*., 3HP, in Hanzlik's experiments (Cerny and Hanzlik, 2006).

Geometrically on **RC** (see Figure 3), the H-O distance along the reaction coordinate is 2.07/2.06 Å for the HS/LS, thus an N-H^…^O hydrogen-bonding interaction exists which might be essential for the following step. Such hydrogen-bonding interaction not only orientates the substrate, therefore facilitating the H-abstraction from the N-H bond, but might weaken the N-H bond in some degree, due to the larger electronegativity of oxygen than nitrogen. On **TS**, the N-H distance (1.15/1.16 Å for the HS/LS) is much shorter than H-O distance (1.36/1.34 Å for the HS/LS), such transition states are asymmetric and exhibit an significant “earlier” characteristic. Similarly in Hirao's work, the transition states of the H-abstraction from the N-H bond were even much earlier, due to its shorter N-H distance (1.04/1.06 Å for the HS/LS) and longer H-O distance (1.76/1.71 Å for the HS/LS).(Hirao et al., 2013a) On the contrary, the H-O distance in P450-catalyzed H-abstraction from the C-H bond was shorter (Ogliaro et al., 2000; de Visser et al., 2002; Kumar et al., 2003; Wang et al., 2006), and thus corresponding to a “later” transition state compared with ours. As expected, the “later” transition state in C-H bond H-abstraction with a higher energy barrier follows the correlation between the transition state and the energy barrier (Shaik et al., 2008; Kumar et al., 2013). Additionally, along the reaction coordinate, the angle of the N-H-O moiety is 167.3°/167.0°For the HS/LS, which is not as linear as the C-H-O moiety in C-H bond H-abstraction. While on **IM**, the newly-formed O-H^…^N hydrogen-bonding interaction with a H-N distance of 1.88/1.89 Å for the HS/LS, becomes stronger than that in **RC**. Such strong hydrogen-bonding interaction, as Rydberg concluded, results in the disadvantage of the following O-rebound process (Rydberg and Olsen, 2011). Besides, the weak nucleophilicity of nitrogen might somehow account for such interference as well. On **TS**_reb_, the Fe-O distance is 1.91/2.00 Å for the HS/LS, whereas the O-N distance is slightly longer (2.15/2.13 Å for the HS/LS). Along the rebound reaction coordinate, the angles of the Fe-O-N moiety is quite different for the quartet (168.9°) and doublet (144.1°) states, the higher energy barrier for the LS of this O-rebound process may be a consequence of such bent **TS** geometry. Such distinct linearities of the Fe-O-N moiety for HS and LS during the O-rebound process were also obtained by Rydberg and Olsen (2011). Whereas, in C-H bond hydroxylations (Ogliaro et al., 2000; de Visser et al., 2002; Kumar et al., 2003; Wang et al., 2006), the transition states of O-rebound process are not as symmetric as those in N-H bond hydroxylations, the Fe-O distance (~1.8 Å) is clearly shorter than the O-C distance (~2.4 Å), and as for the linearity, the rebound transition states in N-H bond hydroxylation are more bent, the angle of Fe-O-C moiety is approximate 158°For the HS. On hydrogen abstraction/ring-opening route, the C_1_-C_3_ (~1.55 Å) distance does not increase until the **IM** species is generated which involves an amino radical. On **TS**_ring_, the C_1_-C_3_ distance increases to 1.90Å for both spin states, however the O-H^…^N hydrogen-bonding distance remains almost permanent, thus indicating the radical substrate is always anchored during the ring-opening process.

**Figure 4:**
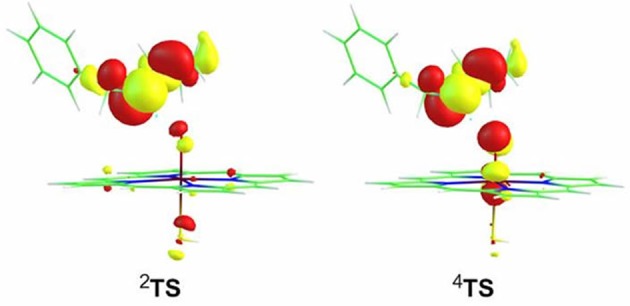
**Single occupied spin-natural orbitals for transition state species of the H-abstraction process**.

Inspection of the spin density (Supplementary Table 2) on **TS**s reveals a confusing result that the distribution on “substrate” is 0.92/-0.88 for the HS/LS, which is suspected exhibiting a PCET character, even if the large KIE values (6.1/5.9 for the HS/LS) supports the HAT mechanism. In retrospect, Hirao et al. obtained the similar tiny energy barrier and distribution on **TS**1A in the favorable *N*-HAT pathway, but they ruled out the involvement of PCET mechanism eventually (Hirao et al., 2013a). Thus, to reveal the genuine mechanism involved in the present H-abstraction from N-H bond, the spin-natural orbitals (SNOs), which are useful for distinguishing between the hydrogen atom transfer (HAT) transition state and the proton-coupled electron transfer transition state, have been analyzed for ^2,4^**TS** species (Figure 4). It is obvious that two π-type lobes on both of the substrate nitrogen atom and the oxidant oxygen atom are perpendicular to the N-H-O axis, which is a typical feature of the PCET transition state. This is similar to the findings of previous studies (Mayer et al., 2002; Usharani et al., 2013; Hirao and Chuanprasit, 2015).

**Figure 5:**
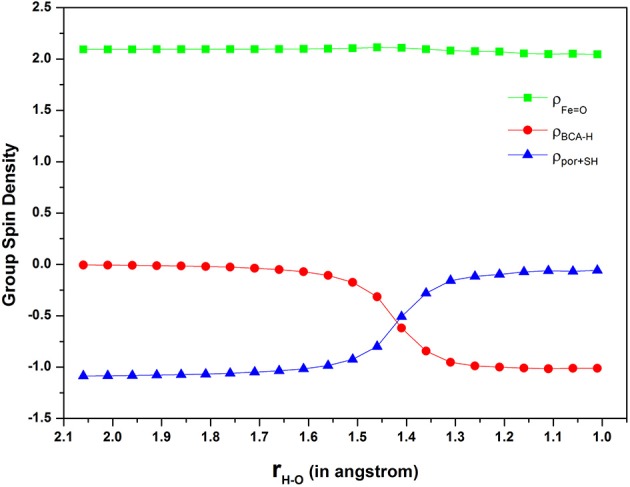
**Diagram of group spin density during the H-abstraction from nitrogen**.

Furthermore, Figure 5 depicts the spin density on ferryl-oxo (green line), “substrate” (red line) and “porpine+thiolate” (blue line) moieties along the reaction coordinate in a stepwise manner. As the hydrogen approaching the oxygen by every 0.05 Å, the spin density on “substrate” is decreasing from 0 to −1 gradually, contrarily, that on “porphyrin+thiolate” is increasing from −1 to 0. Besides, the spin density on ferryl-oxo remains about 2 all along. Inspection of Figure 5, it can be seen that the electron transfer occurs at a H-O distance of 1.4 Å with an obvious change of spin densities on both “substrate” and “porphyrin+thiolate” moieties. From Figure 3, we see that the H-O distance of the transition state is 1.34 Å. This indicates that the electron transfer and the proton transfer are concerted but asynchronous. In other words, the present N-H bond activation should be achieved via a PCET(ET) mechanism as the findings of Usharani et al. (2013).

As mentioned by Cerny and Hanzlik (2005), cytochrome P450 activity was not vanished completely but instead remained 25–30% during the incubation with BCA. Consequently, they attributed this partial inactivation to that BCA reacted with P450 in two reaction pathways (Supplementary Figure 1): a conventional metabolic one on the methylene (-CH_2_-) and cyclopropyl groups, respectively, and a novel inactivation one on the heteroatom, on which our theoretical investigations are based.

### The metabolic pathway

**Figure 6:**
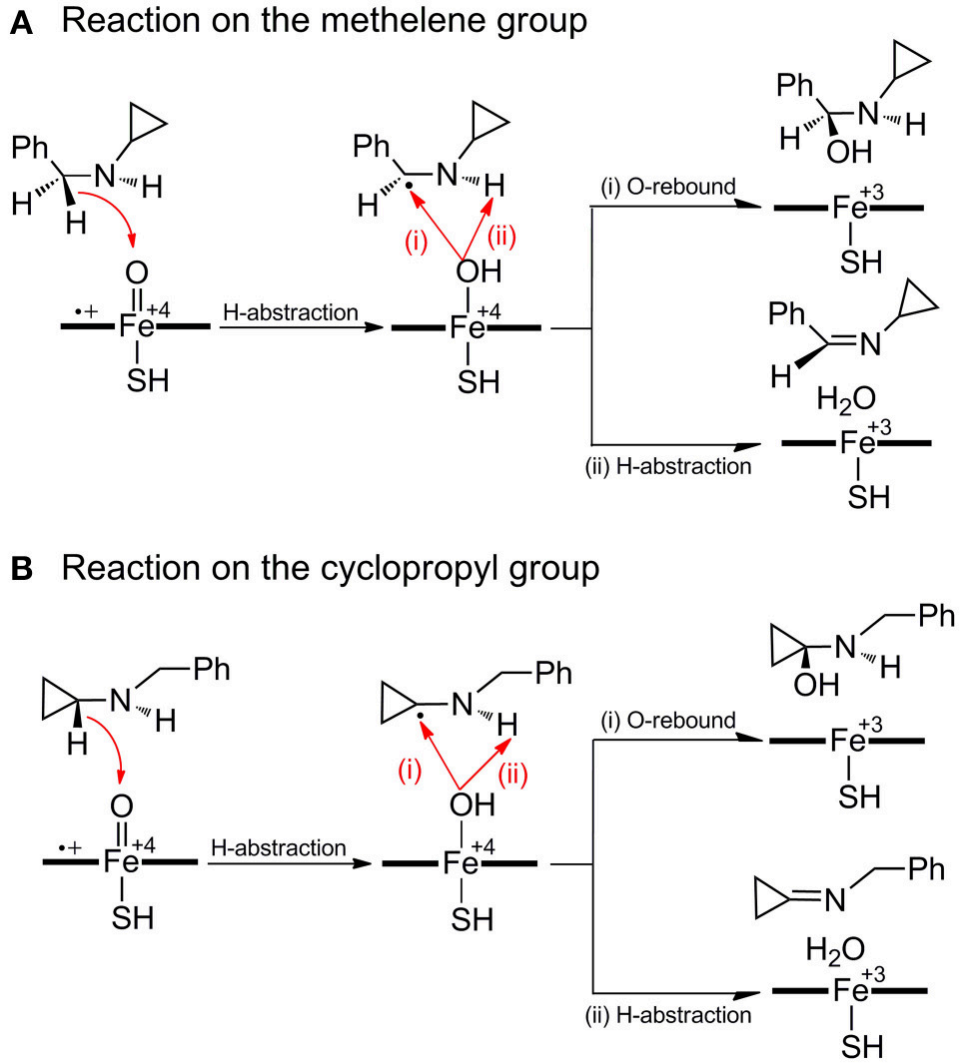
**Proposed mechanisms of hydroxylation and dual hydrogen abstraction (DHA) on both methylene (route A)** and cyclopropyl (route **B**) groups of BCA by P450.

**Figure 7:**
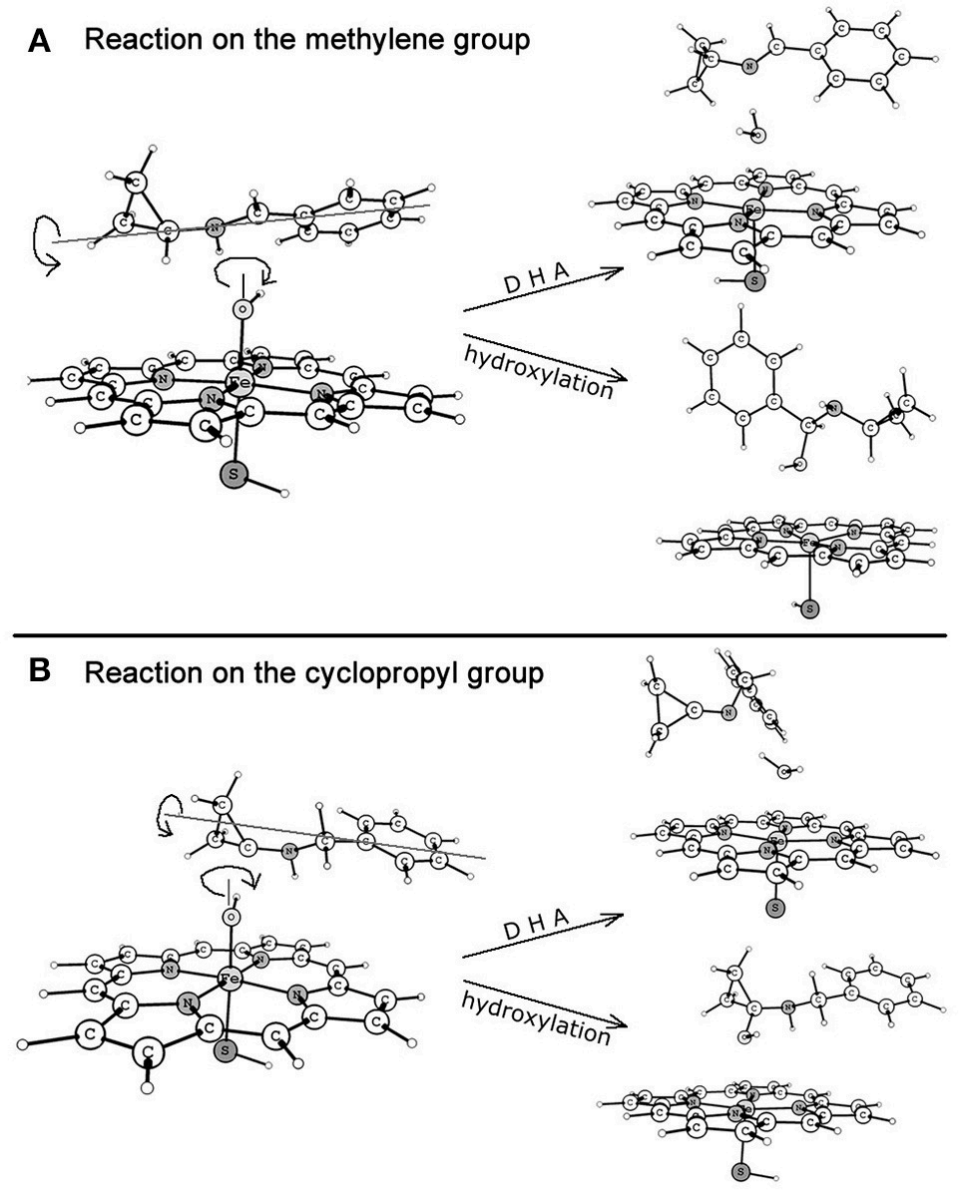
**Dichotomous reaction routes after HAT process on the methylene (route A)** and cyclopropyl (route **B**) groups.

Figure 6 depicts the mechanisms involved in the reactions on the methylene (route A) and cyclopropyl (route B) groups of BCA. Resembling each other, an initial hydrogen atom transfer (HAT) occurs to form the radical and a ferryl-hydroxyl closed-shell porphyrin complex which is unstable. The subtle geometric feature of the radical moiety is responsible for the dichotomous behaviors of the reactions in the second step, which are determined by the action of the radical or the newly-generated hydroxyl group as shown in Figure 7. Specifically, when the hydroxyl rotates independently, it will generate carbinolamine via a direct O-rebound process, while the rotation of the hydroxyl and the radical is coupled, an unexpected enamine product will be produced via another HAT process from the adjacent heteroatom (the overall reaction is a dual hydrogen abstraction process, DHA). The competitive DHA mechanism is also a conventional mechanism involved in the P450-catalyzed cholesterol side-chain cleavage reaction and other oxidations (Vanlier and Rousseau, 1976; Sono et al., 1996; Kumar et al., 2004; Oh et al., 2005; Wang et al., 2007b; Ji et al., 2015).

**Figure 8:**
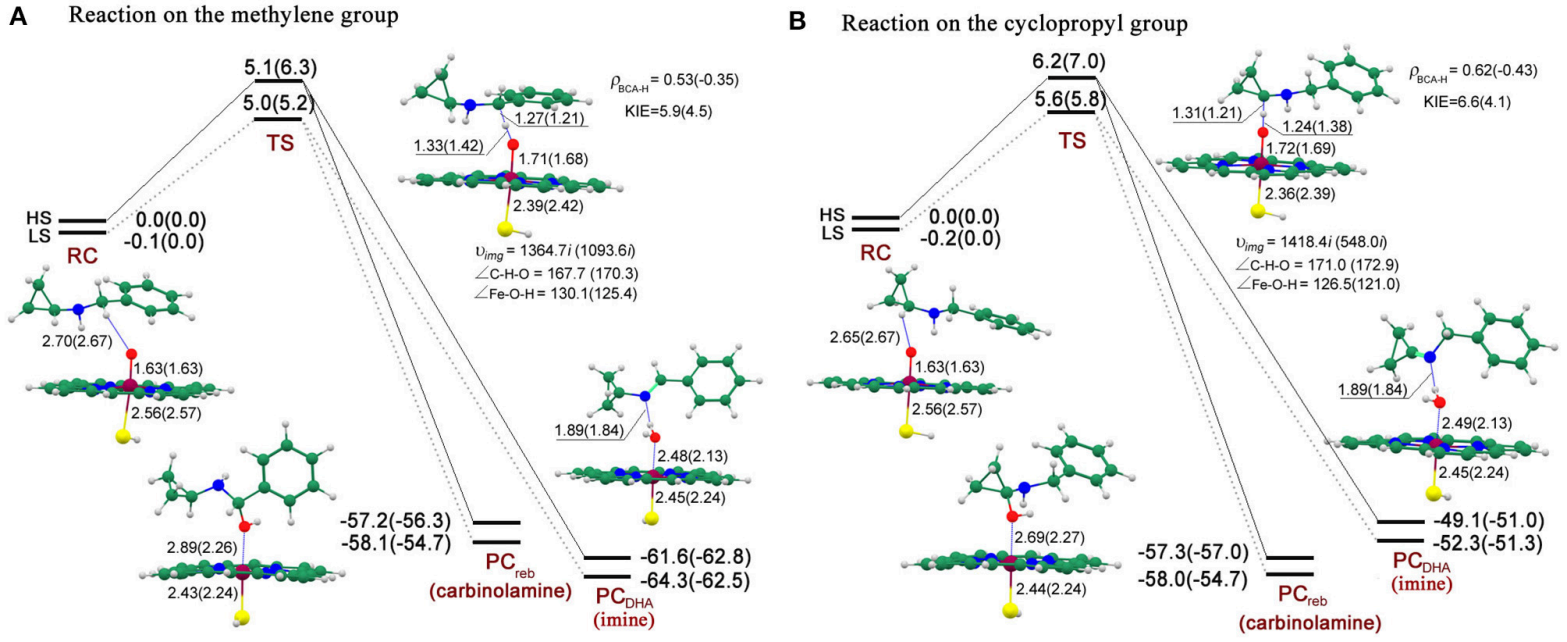
**Energy profiles (UB3LYP/B2//B1) for both reactions on the methylene (route A)** and cyclopropyl (route **B**) groups at the E1(E2) level and the geometries (UB3LYP/B1) of the key intermediates for the HS(LS) involved. The relative energy is in kcal/mol, bond length is in Å, bond angle is in degree and the imaginary frequency is in cm^−1^ unit.

Figure 8 shows the energy profiles for both reactions on routes A and B accompanied by the geometries of the key intermediates involved. On **RC**, the high-spin quartet state (HS) and the low-spin doublet state (LS) are nascent from the degenerate states of CpdI, with the LS lying 0.1/0.2 kcal/mol lower under the HS on route a/b in the gas phase. The energy differences are eliminated by the inclusion of bulk polar effect.

On route A, the energy barrier of the rate-limiting step, which refers to HAT process, is 5.1/5.0 kcal/mol for the HS/LS in the gas phase, which increases to 6.3/5.2 kcal/mol when bulk polar effect is included. As discussed above, the following step is competitive and depends on the rotation of the hydroxyl and the radical. Once after the barrierless O-rebound process, the carbinolamine (−57.2/−58.1 kcal/mol for the HS/LS) is produced, which might subsequently decompose to benzaldehyde and cyclopropylamine, probably in a non-enzymatic environment assisted by water molecule. By contrast, the DHA process, in which the second H-abstraction from nitrogen atom is also barrierless, is a more exothermic process due to the lower energy (−61.6/−64.3 kcal/mol for the HS/LS) of enamine product complex (**PC**). Along the C-H-O reaction coordinate, the C-H distance is 1.27/1.21 Å for the HS/LS which is shorter than 1.33/1.42 Å of the H-O distance, and consequently making ^2^**TS** an earlier transition state compared to ^4^**TS**. Inspection of the spin density (Supplementary Table 6) reveals that, for the HS/LS, the spin density populated on “substrate” (BCA-H moiety) is 0.53/−0.35, while “porphyrin+thiolate” moiety holds 0.45/−0.48. Thus, the **TS** species are deemed to exhibit an “HAT-type” character. In addition, the semi-classical Eyring KIE value (see Computational methods above) for each state is 5.9/4.5, which is relatively large and supports the HAT mechanism.

Whereas on route B, there are still a few discrepancies compared to those on the similar route A. The energy barrier of the rate-limiting step (6.2/5.7 kcal/mol in the gas phase and 7.0/5.8 kcal/mol in solvent for the HS/LS) is about 1 kcal/mol higher than that on route A. However, given that the tiny difference of the energy barriers between these two reactions, they probably occur competitively. Alternatively on route B, the carbinolamine product is generated via a more exothermic HAT/O-rebound process compared to the competitive DHA process. The carbinolamine from this route would undergo following decomposition to yield cyclopropanone and benzylamine identified in Hanzlik's experiments (Cerny and Hanzlik, 2005, 2006). Geometrically, the ^4^**TS** species is the only one that possesses a “later” transition state geometry in both reactions, in which C-H distance (1.31 Å) is longer than H-O distance (1.24 Å).

## Conclusion

For decades, the mechanism-based inactivation role of *N*-benzyl-*N*-cyclopropylamine to cytochrome P450 has been attracting great interests. Theoretical investigation on the way they function is contributory to clinical drug design. Thereby we performed DFT calculation on P450-catalyzed BCA reaction, in which it proceeded on dichotomous metabolic and inactivation pathways. In metabolic pathway, besides the carbinolamine, which is the precursor to the product identified experimentally, an unexpected enamine product was formed via the competitive DHA route switched by the coupled rotation of the radical and hydroxyl group. In inactivation pathway, the SET process was invalidated for its high energy barrier. Whereas, an amino radical was formed after the initial H-abstraction from nitrogen atom, the energy barrier involved was 0.6/0.4 kcal/mol for two spin states, and then dichotomous behaviors were encountered again. Owing to the steric hindrance caused by the hydrogen-bonding between O-H and N on **IM**, the reaction would primarily proceed through the rapid ring-opening rather than the O-rebound process to generate a *C*-centered radical species. Such carbon radical species may not only subsequently convert to 3HP, which was identified as the major product experimentally, but essentially account for the inactivation by covalent binding to amino residue. Intriguingly, in addition to the extremely low energy barrier of the H-abstraction in inactivation pathway, the spin density distribution on “substrate” moiety is approaching ±1, exhibiting several PCET characters. Furthermore, the SNOs for the transition state in such H-abstraction together with the analysis of spin densities on “substrate” and “porpine+thiolate” moieties along the reaction coordinate definitely demonstrate a PCET(ET) mechanism.

## Author contributions

All authors listed, have made substantial, direct and intellectual contribution to the work, and approved it for publication.

### Conflict of interest statement

The authors declare that the research was conducted in the absence of any commercial or financial relationships that could be construed as a potential conflict of interest.
