# Supplementary material for: Suicide Inhibition of Cytochrome P450 Enzymes by Cyclopropylamines via a Ring-Opening Mechanism: Proton-Coupled Electron Transfer Makes a Difference
Source: Front Chem. 2017 Jan 31;5:3. doi: 10.3389/fchem.2017.00003 (PMC5281577; doi:10.3389/fchem.2017.00003)
Supplement: Supplementary file 1 [file DataSheet1.DOCX]

Supplementary Material

Suicide Inhibition of Cytochrome P450 Enzymes by Cyclopropylamines via a Ring-opening Mechanism: Proton-Coupled Electron Transfer Makes a Difference

Xiaoqian Zhang^2^, Xiao-Xi Li^1*^, Yufang Liu^2^, Yong Wang^1*^

*** Correspondence:** Yong Wang: wangyong@licp.cas.cn; Xiao-Xi Li: lixiaoxi@licp.cas.cn

## Supplementary Figures

**

**

**Supplementary Figure 1.** Proposed reaction pathways of *N*-benzyl-*N*-cyclopropylamine (BCA) catalyzed by P450.





**Supplementary Figure 2.** Orbital diagram for single electron transfer on the quartet (doublet) state along the hydrogen abstraction from N-H bond.


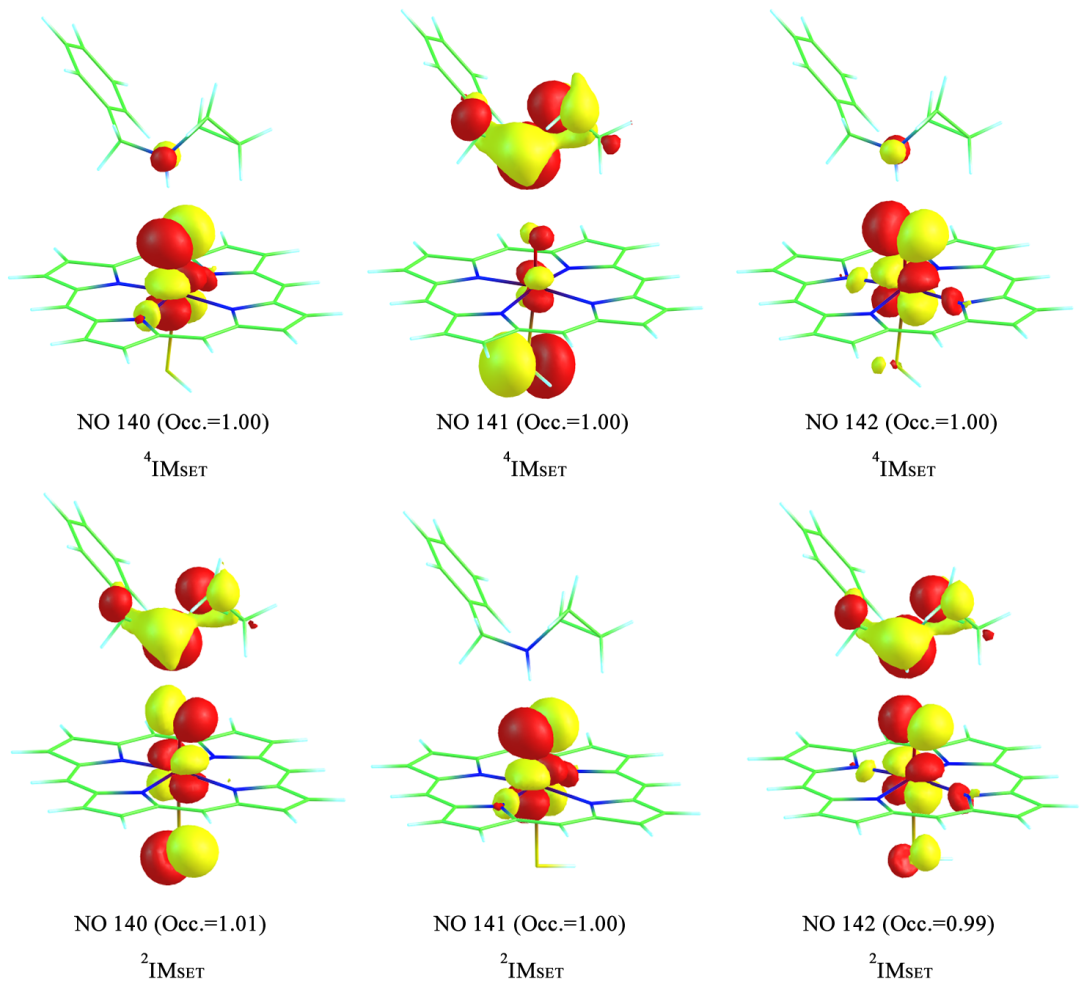


**Supplementary Figure 3.** Single occupied natural orbitals of the doublet species obtained along the reaction coordinate by every 0.05 Å in the hydrogen abstraction process calculated at the UB3LYP/B1 level. To be continued.


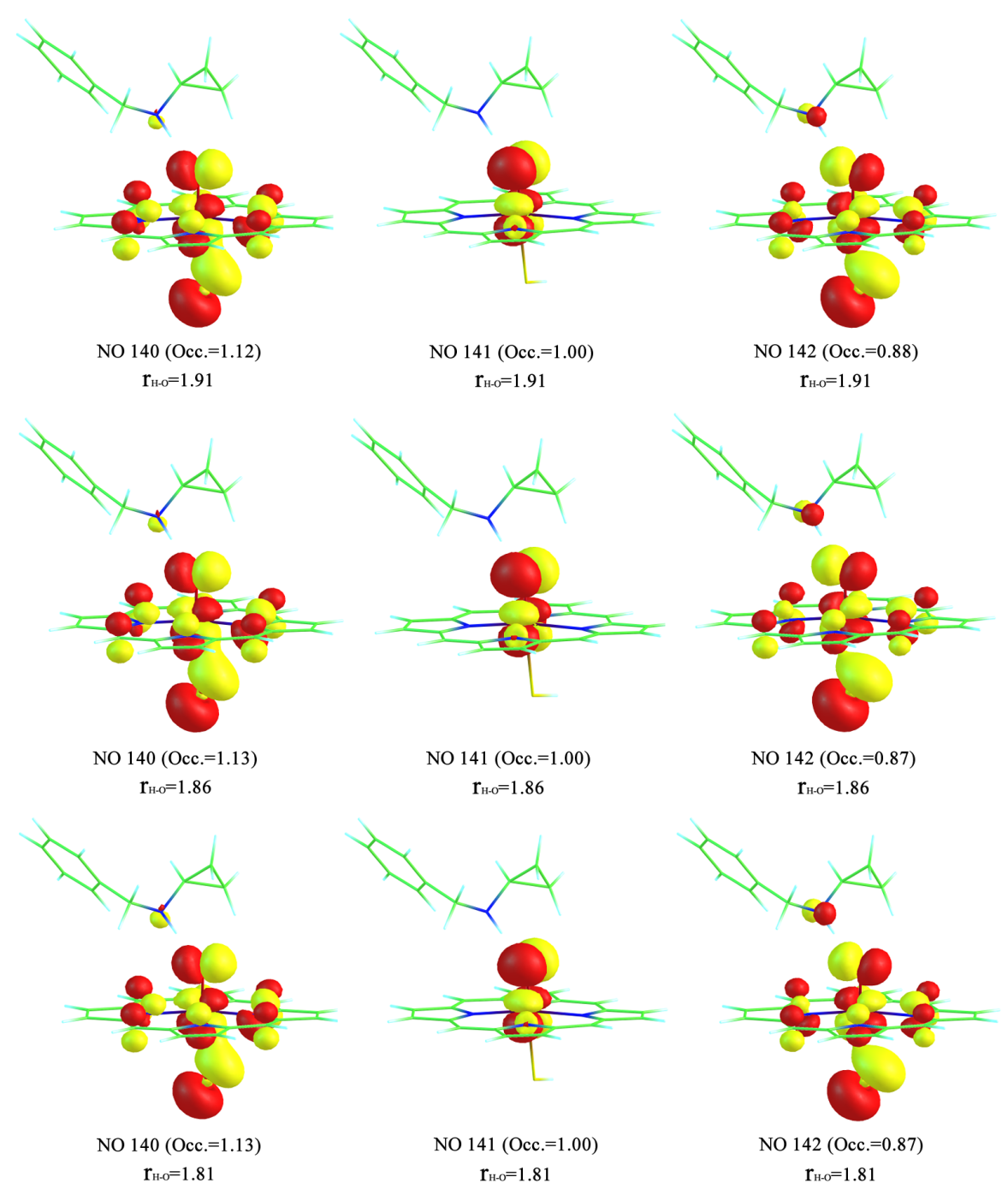


**Supplementary Figure 3.** Single occupied natural orbitals of the doublet species obtained along the reaction coordinate by every 0.05 Å in the hydrogen abstraction process calculated at the UB3LYP/B1 level. To be continued.


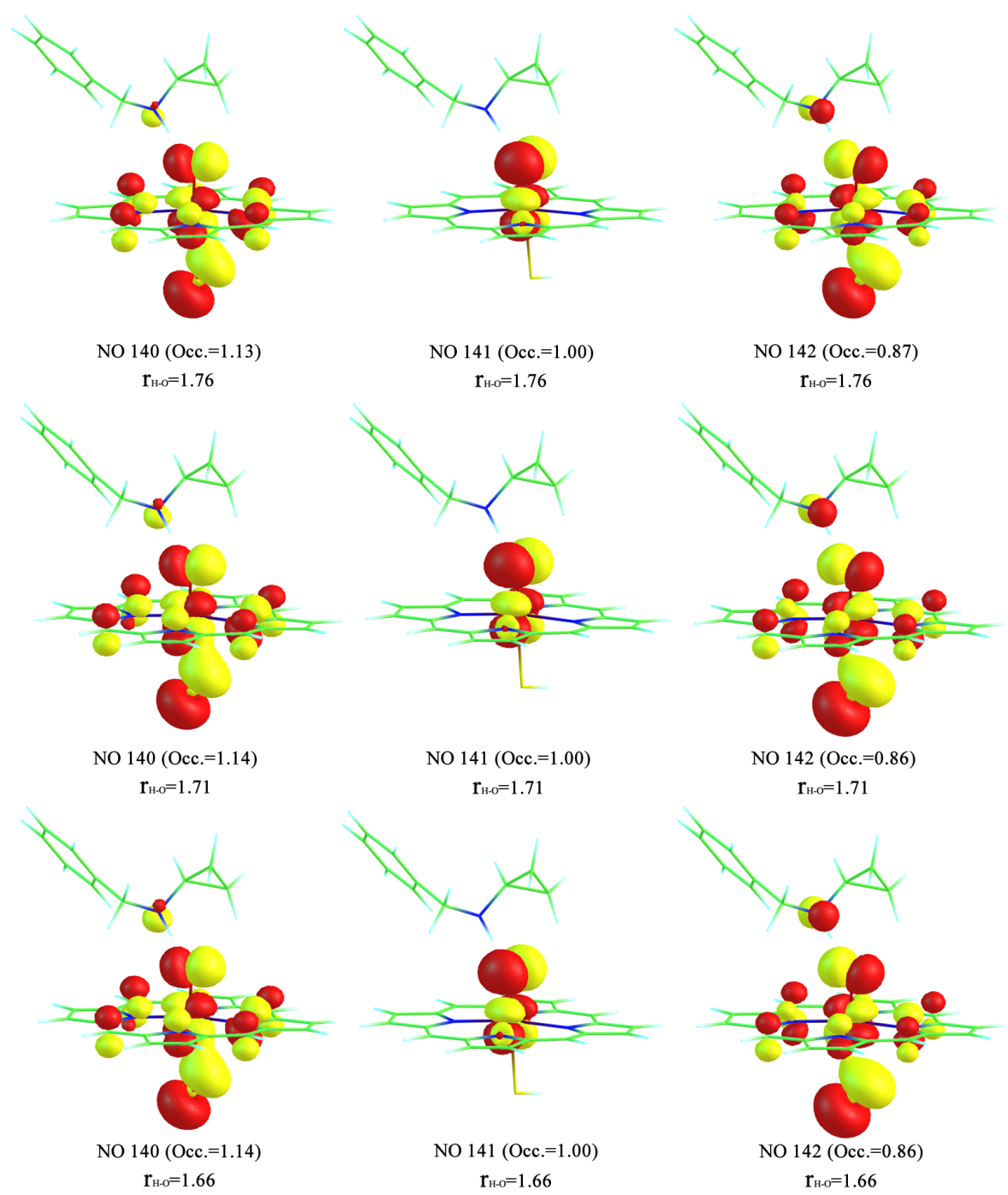


**Supplementary Figure 3.** Single occupied natural orbitals of the doublet species obtained along the reaction coordinate by every 0.05 Å in the hydrogen abstraction process calculated at the UB3LYP/B1 level. To be continued.


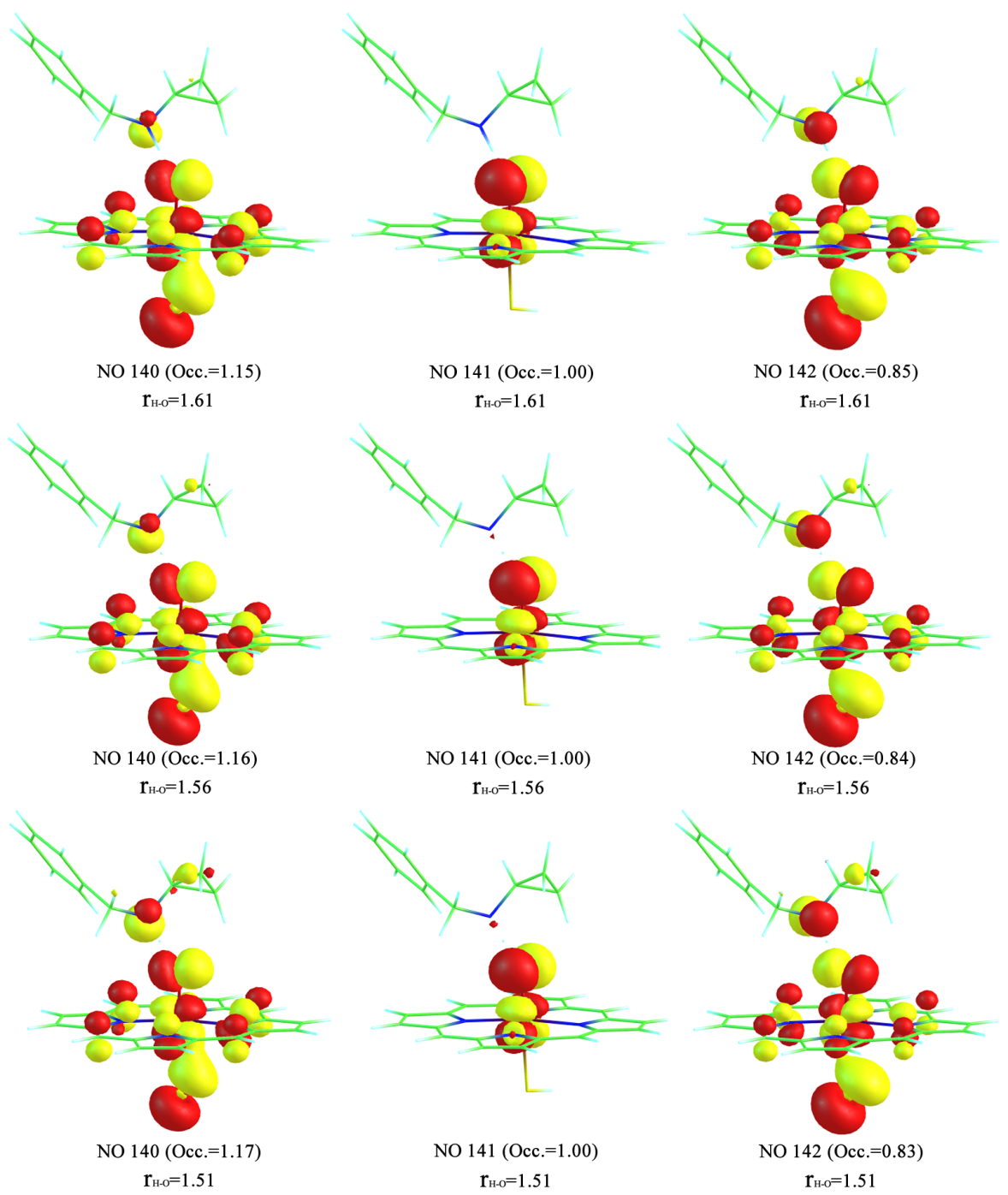


**Supplementary Figure 3.** Single occupied natural orbitals of the doublet species obtained along the reaction coordinate every 0.05 Å in the hydrogen abstraction process calculated at the UB3LYP/B1 level. To be continued.


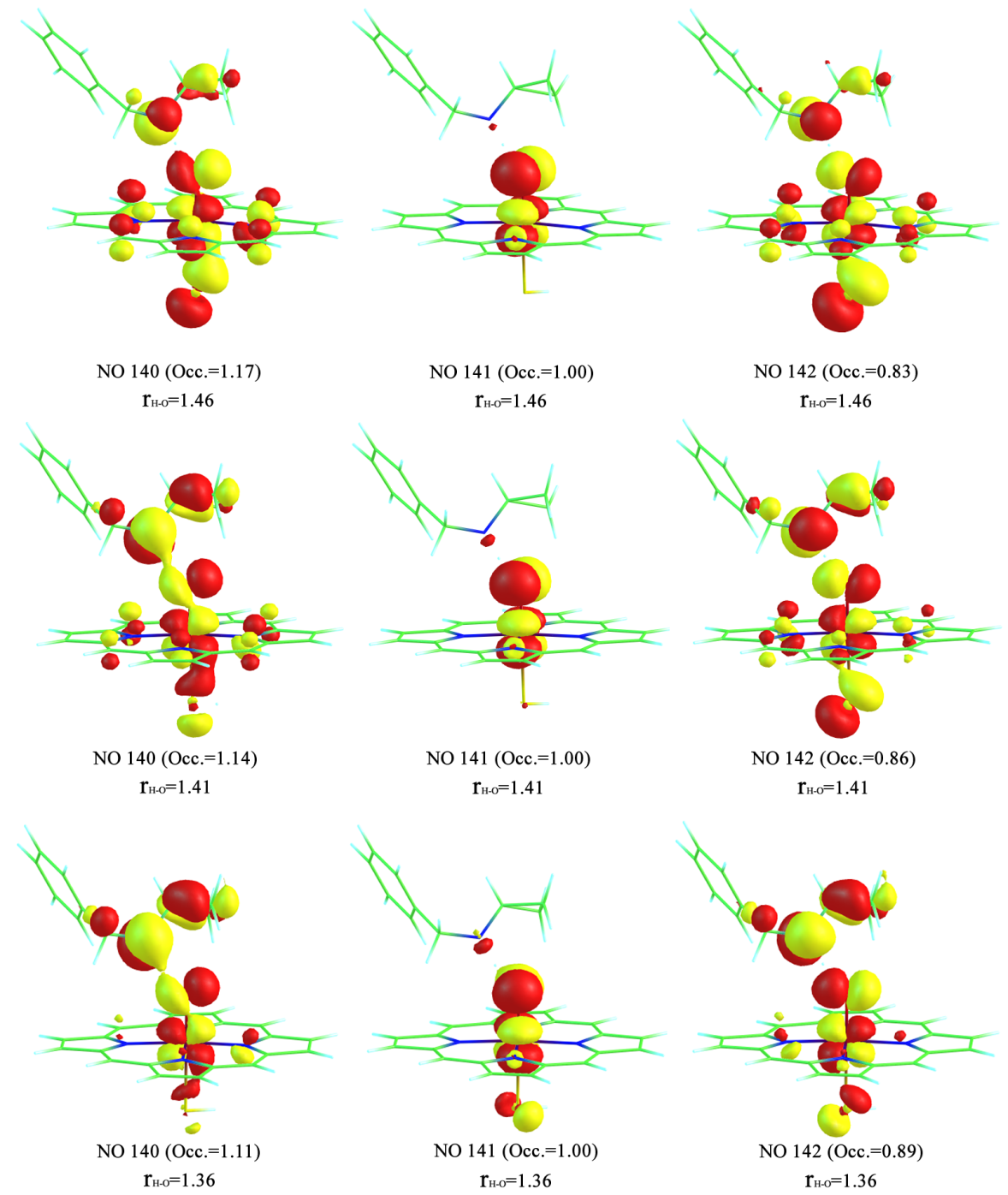


**Supplementary Figure 3.** Single occupied natural orbitals of the doublet species obtained along the reaction coordinate by every 0.05 Å in the hydrogen abstraction process calculated at the UB3LYP/B1 level.

## Supplementary Tables

**Supplementary Table 1.** SCF energies at various computational levels for hydrogen abstraction/ring-opening and hydrogen abstraction/O-rebound processes of BCA.

|  | OPT | | OPT+ZPE | | SPE | | SPE+ZPE | | SOLV | | SOLV+ZPE | | G(Free Energy) | |
| --- | --- | --- | --- | --- | --- | --- | --- | --- | --- | --- | --- | --- | --- | --- |
|  | ***E*** *^a^* | **Δ*E*** | ***E*** | **Δ*E*** | ***E*** | **Δ*E*** | ***E*** | **Δ*E*** | ***E*** | **Δ*E*** | ***E*** | **Δ*E*** | ***G*** | **Δ*G*** |
| ^4^**RC** | -2029.389543 | **0.0** | -2028.892396 | **0.0** | -2029.861883 | **0.0** | -2029.364736 | **0.0** | -2029.885630 | **0.0** | -2029.388483 | **0.0** | -2028.964363 | **0.0** |
| ^2^**RC** | -2029.389656 | **-0.1** | -2028.892660 | **-0.2** | -2029.862184 | **-0.2** | -2029.365188 | **-0.3** | -2029.885566 | **0.0** | -2029.388570 | **-0.1** | -2028.964792 | **-0.3** |
| ^4^**TS** | -2029.376628 | **8.1** | -2028.885238 | **4.5** | -2029.855208 | **4.2** | -2029.363818 | **0.6** | -2029.884523 | **0.7** | -2029.393133 | **-2.9** | -2028.953403 | **6.9** |
| ^2^**TS** | -2029.376712 | **8.1** | -2028.885415 | **4.4** | -2029.855459 | **4.0** | -2029.364162 | **0.4** | -2029.884083 | **1.0** | -2029.392786 | **-2.7** | -2028.953095 | **7.1** |
| ^4^**IM** | -2029.390631 | **-0.7** | -2028.895470 | **-1.9** | -2029.872216 | **-6.5** | -2029.377055 | **-7.7** | -2029.893935 | **-5.2** | -2029.398774 | **-6.5** | -2028.965294 | **-0.6** |
| ^2^**IM** | -2029.390486 | **-0.6** | -2028.895278 | **-1.8** | -2029.872474 | **-6.6** | -2029.377266 | **-7.9** | -2029.892917 | **-4.6** | -2029.397709 | **-5.8** | -2028.964243 | **0.1** |
| ^4^**TS**_reb_ | -2029.370293 | **12.1** | -2028.876703 | **9.8** | -2029.854447 | **4.7** | -2029.360857 | **2.4** | -2029.876276 | **5.9** | -2029.382686 | **3.6** | -2028.945752 | **11.7** |
| ^2^**TS**_reb_ | -2029.367617 | **13.8** | -2028.873224 | **12.0** | -2029.853451 | **5.3** | -2029.359058 | **3.6** | -2029.875498 | **6.4** | -2029.381105 | **4.6** | -2028.939873 | **15.4** |
| ^4^**TS**_ring_ | -2029.380340 | **5.8** | -2028.887246 | **3.2** | -2029.863540 | **-1.0** | -2029.370446 | **-3.6** | -2029.884685 | **0.6** | -2029.391591 | **-2.0** | -2028.957183 | **4.5** |
| ^2^**TS**_ring_ | -2029.380304 | **5.8** | -2028.887250 | **3.2** | -2029.863548 | **-1.0** | -2029.370494 | **-3.6** | -2029.884428 | **0.8** | -2029.391374 | **-1.8** | -2028.957104 | **4.6** |
| ^4^**PC**_reb_ | -2029.420078 | **-19.2** | -2028.922810 | **-19.1** | -2029.899440 | **-23.6** | -2029.402172 | **-23.5** | -2029.924656 | **-24.5** | -2029.427388 | **-24.4** | -2028.995616 | **-19.6** |
| ^2^**PC**_reb_ | -2029.424242 | **-21.8** | -2028.925547 | **-20.8** | -2029.900539 | **-24.3** | -2029.401844 | **-23.3** | -2029.919496 | **-21.3** | -2029.420801 | **-20.3** | -2028.991519 | **-17.0** |
| ^4^**PC**_ring_ | -2029.394257 | **-3.0** | -2028.901479 | **-5.7** | -2029.878832 | **-10.6** | -2029.386054 | **-13.4** | -2029.901835 | **-10.2** | -2029.409057 | **-12.9** | -2028.971794 | **-4.7** |
| ^2^**PC**_ring_ | -2029.394312 | **-3.0** | -2028.901567 | **-5.8** | -2029.878893 | **-10.7** | -2029.386148 | **-13.4** | -2029.901704 | **-10.1** | -2029.408959 | **-12.8** | -2028.971321 | **-4.4** |

*^a^* Absolute SCF energies ***E*** are in a.u. and relative SCF energies **Δ*E*** are in kcal/mol.

**Supplementary Table 2.** The Mulliken spin density and charge of various reaction species for hydrogen abstraction/ring-opening and hydrogen abstraction/O-rebound processes of BCA calculated at the B3LYP/B1 level.

|  | Spin density | | | | | |  | Charge | | | | | |
| --- | --- | --- | --- | --- | --- | --- | --- | --- | --- | --- | --- | --- | --- |
|  | Fe | O | por | SH | H | rest |  | Fe | O | por | SH | H | rest |
| ^4^**RC** | 1.14 | 0.88 | 0.46 | 0.50 | 0.00 | 0.02 |  | 0.48 | -0.44 | 0.00 | -0.04 | 0.37 | -0.37 |
| ^2^**RC** | 1.26 | 0.83 | -0.53 | -0.55 | 0.00 | -0.01 |  | 0.48 | -0.44 | -0.01 | -0.04 | 0.37 | -0.37 |
| ^4^**TS** | 1.48 | 0.59 | -0.03 | 0.06 | -0.02 | 0.92 |  | 0.40 | -0.64 | -0.32 | -0.10 | 0.49 | 0.17 |
| ^2^**TS** | 1.56 | 0.54 | -0.18 | -0.05 | 0.01 | -0.88 |  | 0.41 | -0.65 | -0.32 | -0.10 | 0.50 | 0.16 |
| ^4^**IM** | 1.73 | 0.30 | -0.12 | 0.09 | 0.00 | 1.00 |  | 0.39 | -0.70 | -0.24 | 0.01 | -0.13 | 0.67 |
| ^2^**IM** | 1.72 | 0.30 | -0.12 | 0.09 | 0.00 | -0.99 |  | 0.39 | -0.71 | -0.24 | 0.01 | -0.13 | 0.67 |
| ^4^**TS**_reb_ | 2.21 | -0.10 | -0.10 | 0.29 | 0.00 | 0.70 |  | 0.44 | -0.64 | -0.32 | -0.07 | -0.13 | 0.72 |
| ^2^**TS**_reb_ | 0.87 | 0.26 | 0.32 | 0.29 | 0.00 | -0.75 |  | 0.30 | -0.65 | -0.21 | -0.03 | -0.13 | 0.71 |
| ^4^**TS**_ring_ | 1.72 | 0.31 | -0.12 | 0.08 | 0.00 | 1.00 |  | 0.39 | -0.71 | -0.26 | 0.01 | -0.13 | 0.69 |
| ^2^**TS**_ring_ | 1.72 | 0.31 | -0.12 | 0.09 | 0.00 | -0.99 |  | 0.39 | -0.71 | -0.26 | 0.01 | -0.13 | 0.69 |
| ^4^**PC**_reb_ | 2.46 | 0.00 | 0.06 | 0.48 | 0.00 | 0.00 |  | 0.44 | -0.56 | -0.33 | -0.15 | -0.13 | 0.73 |
| ^2^**PC**_reb_ | 1.11 | 0.00 | -0.09 | -0.02 | 0.00 | 0.00 |  | 0.25 | -0.51 | -0.44 | 0.03 | -0.13 | 0.79 |
| ^4^**PC**_ring_ | 1.72 | 0.31 | -0.12 | 0.08 | 0.00 | 1.00 |  | 0.40 | -0.70 | -0.26 | 0.01 | -0.13 | 0.68 |
| ^2^**PC**_ring_ | 1.72 | 0.31 | -0.12 | 0.08 | 0.00 | -0.99 |  | 0.40 | -0.70 | -0.26 | 0.01 | -0.13 | 0.68 |

**Supplementary Table 3.** The Mulliken spin density and charge of the SET intermediate species calculated at the B3LYP/B1 level.

|  | Spin density | | | | | |  | Charge | | | | | |
| --- | --- | --- | --- | --- | --- | --- | --- | --- | --- | --- | --- | --- | --- |
|  | Fe | O | por | SH | H | rest |  | Fe | O | por | SH | H | rest |
| ^4^**IM**_SET_ | 1.28 | 0.76 | 0.01 | 0.24 | -0.01 | 0.73 |  | 0.49 | -0.55 | -0.40 | -0.19 | 0.44 | 0.20 |
| ^2^**IM**_SET_ | 1.33 | 0.77 | -0.07 | -0.22 | 0.01 | -0.83 |  | 0.49 | -0.56 | -0.44 | -0.24 | 0.45 | 0.30 |

**Supplementary Table 4.** The Mulliken spin density and charge of the doublet species obtained along the reaction coordinate by every 0.05 Å in the hydrogen abstraction from N-H bond calculated at the UB3LYP/B1 level.

| r_O-H_ (in Å) | Spin density | | | | | |  | Charge | | | | | |
| --- | --- | --- | --- | --- | --- | --- | --- | --- | --- | --- | --- | --- | --- |
|  | Fe | O | por | SH | H | rest |  | Fe | O | por | SH | H | rest |
| 2.06 | 1.27 | 0.83 | -0.53 | -0.55 | 0.00 | -0.01 |  | 0.48 | -0.44 | -0.01 | -0.04 | 0.37 | -0.37 |
| 2.01 | 1.27 | 0.82 | -0.53 | -0.55 | 0.00 | -0.01 |  | 0.48 | -0.45 | -0.01 | -0.04 | 0.38 | -0.37 |
| 1.96 | 1.27 | 0.82 | -0.53 | -0.55 | 0.00 | -0.01 |  | 0.49 | -0.45 | -0.01 | -0.04 | 0.38 | -0.38 |
| 1.91 | 1.28 | 0.81 | -0.54 | -0.54 | 0.00 | -0.01 |  | 0.48 | -0.45 | 0.00 | -0.04 | 0.39 | -0.38 |
| 1.86 | 1.29 | 0.81 | -0.54 | -0.54 | 0.00 | -0.02 |  | 0.48 | -0.46 | 0.00 | -0.04 | 0.39 | -0.38 |
| 1.81 | 1.29 | 0.80 | -0.53 | -0.53 | 0.00 | -0.02 |  | 0.48 | -0.46 | 0.00 | -0.04 | 0.40 | -0.38 |
| 1.76 | 1.30 | 0.79 | -0.53 | -0.53 | 0.00 | -0.03 |  | 0.48 | -0.47 | 0.00 | -0.04 | 0.40 | -0.38 |
| 1.71 | 1.31 | 0.78 | -0.53 | -0.52 | 0.00 | -0.04 |  | 0.48 | -0.48 | -0.01 | -0.04 | 0.41 | -0.37 |
| 1.66 | 1.33 | 0.77 | -0.53 | -0.51 | -0.01 | -0.06 |  | 0.48 | -0.48 | -0.01 | -0.04 | 0.41 | -0.36 |
| 1.61 | 1.34 | 0.76 | -0.52 | -0.50 | -0.01 | -0.08 |  | 0.48 | -0.49 | -0.01 | -0.04 | 0.42 | -0.35 |
| 1.56 | 1.37 | 0.73 | -0.50 | -0.48 | -0.01 | -0.11 |  | 0.48 | -0.51 | -0.03 | -0.05 | 0.43 | -0.33 |
| 1.51 | 1.40 | 0.71 | -0.48 | -0.44 | 0.00 | -0.18 |  | 0.47 | -0.52 | -0.05 | -0.06 | 0.44 | -0.29 |
| 1.46 | 1.46 | 0.66 | -0.43 | -0.36 | 0.00 | -0.31 |  | 0.46 | -0.55 | -0.09 | -0.07 | 0.45 | -0.20 |
| 1.41 | 1.52 | 0.59 | -0.32 | -0.19 | 0.01 | -0.61 |  | 0.43 | -0.60 | -0.20 | -0.10 | 0.48 | 0.00 |
| 1.36 | 1.55 | 0.55 | -0.20 | -0.07 | 0.01 | -0.83 |  | 0.41 | -0.64 | -0.30 | -0.10 | 0.49 | 0.13 |

**Supplementary Table 5.** SCF energies at various computational levels for C-H hydroxylation and dual hydrogen abstraction on the methylene group of BCA.

|  | OPT | | OPT+ZPE | | SPE | | SPE+ZPE | | SOLV | | SOLV+ZPE | | G(Free Energy) | |
| --- | --- | --- | --- | --- | --- | --- | --- | --- | --- | --- | --- | --- | --- | --- |
|  | ***E****^a^* | **Δ*E*** | ***E*** | **Δ*E*** | ***E*** | **Δ*E*** | ***E*** | **Δ*E*** | ***E*** | **Δ*E*** | ***E*** | **Δ*E*** | ***G*** | **Δ*G*** |
| ^4^**RC** | -2029.389736 | **0.0** | -2028.892579 | **0.0** | -2029.861473 | **0.0** | -2029.364316 | **0.0** | -2029.885762 | **0.0** | -2029.388605 | **0.0** | -2028.964060 | **0.0** |
| ^2^**RC** | -2029.389983 | **-0.2** | -2028.892861 | **-0.2** | -2029.861643 | **-0.1** | -2029.364521 | **-0.1** | -2029.885695 | **0.0** | -2029.388573 | **0.0** | -2028.962670 | **0.9** |
| ^4^**TS** | -2029.369672 | **12.6** | -2028.877727 | **9.3** | -2029.848068 | **8.4** | -2029.356123 | **5.1** | -2029.870559 | **9.5** | -2029.378614 | **6.3** | -2028.943797 | **12.7** |
| ^2^**TS** | -2029.371627 | **11.4** | -2028.879239 | **8.4** | -2029.848711 | **8.0** | -2029.356323 | **5.0** | -2029.872713 | **8.2** | -2029.380325 | **5.2** | -2028.944931 | **12.0** |
| ^4^**PC**_reb_ | -2029.472883 | **-52.2** | -2028.973944 | **-51.1** | -2029.954334 | **-58.3** | -2029.455395 | **-57.2** | -2029.977238 | **-57.4** | -2029.478299 | **-56.3** | -2029.044307 | **-50.4** |
| ^2^**PC**_reb_ | -2029.478089 | **-55.4** | -2028.977829 | **-53.5** | -2029.957115 | **-60.0** | -2029.456855 | **-58.1** | -2029.976048 | **-56.7** | -2029.475788 | **-54.7** | -2029.042871 | **-49.5** |
| ^4^**PC**_DHA_ | -2029.469262 | **-49.9** | -2028.974343 | **-51.3** | -2029.957446 | **-60.2** | -2029.462527 | **-61.6** | -2029.983677 | **-61.4** | -2029.488758 | **-62.8** | -2029.042871 | **-49.5** |
| ^2^**PC**_DHA_ | -2029.480051 | **-56.7** | -2028.982996 | **-56.7** | -2029.963781 | **-64.2** | -2029.466726 | **-64.3** | -2029.985277 | **-62.4** | -2029.488222 | **-62.5** | -2029.050718 | **-54.4** |

*^a^* Absolute SCF energies ***E*** are in a.u. and relative SCF energies **Δ*E*** are in kcal/mol.

**Supplementary Table 6.** The Mulliken spin density and charge of various reaction species for C-H hydroxylation and dual hydrogen abstraction on the methylene group of BCA calculated at the B3LYP/B1 level.

|  | Spin density | | | | | |  | Charge | | | | | |
| --- | --- | --- | --- | --- | --- | --- | --- | --- | --- | --- | --- | --- | --- |
|  | Fe | O | por | SH | H | rest |  | Fe | O | por | SH | H | rest |
| ^4^**RC** | 1.10 | 0.92 | 0.45 | 0.53 | 0.00 | 0.01 |  | 0.49 | -0.40 | -0.02 | -0.05 | 0.17 | -0.19 |
| ^2^**RC** | 1.21 | 0.87 | -0.51 | -0.58 | 0.00 | 0.00 |  | 0.49 | -0.41 | -0.03 | -0.04 | 0.17 | -0.19 |
| ^4^**TS** | 1.30 | 0.73 | 0.10 | 0.35 | -0.01 | 0.53 |  | 0.42 | -0.55 | -0.25 | -0.01 | 0.35 | 0.04 |
| ^2^**TS** | 1.54 | 0.31 | -0.28 | -0.20 | -0.02 | -0.35 |  | 0.42 | -0.53 | -0.20 | -0.06 | 0.32 | 0.06 |
| ^4^**PC**_reb_ | 2.47 | 0.00 | 0.05 | 0.48 | 0.00 | 0.00 |  | 0.45 | -0.64 | -0.34 | -0.15 | 0.43 | 0.25 |
| ^2^**PC**_reb_ | 1.09 | 0.00 | -0.09 | -0.01 | 0.00 | 0.00 |  | 0.26 | -0.62 | -0.44 | 0.04 | 0.42 | 0.34 |
| ^4^**PC**_DHA_ | 2.54 | 0.02 | -0.02 | 0.45 | 0.00 | 0.00 |  | 0.45 | -0.78 | -0.42 | -0.18 | 0.42 | 0.51 |
| ^2^**PC**_DHA_ | 1.05 | 0.00 | -0.08 | 0.03 | 0.00 | 0.00 |  | 0.25 | -0.78 | -0.46 | 0.01 | 0.44 | 0.55 |

**Supplementary Table 7.** SCF energies at various computational levels for C-H hydroxylation and dual hydrogen abstraction on the cyclopropyl group of BCA.

|  | OPT | | OPT+ZPE | | SPE | | SPE+ZPE | | SOLV | | SOLV+ZPE | | G(Free Energy) | |
| --- | --- | --- | --- | --- | --- | --- | --- | --- | --- | --- | --- | --- | --- | --- |
|  | ***E****^a^* | **Δ*E*** | ***E*** | **Δ*E*** | ***E*** | **Δ*E*** | ***E*** | **Δ*E*** | ***E*** | **Δ*E*** | ***E*** | **Δ*E*** | ***G*** | **Δ*G*** |
| ^4^**RC** | -2029.389634 | **0.0** | -2028.892461 | **0.0** | -2029.861272 | **0.0** | -2029.364099 | **0.0** | -2029.885168 | **0.0** | -2029.387995 | **0.0** | -2028.964017 | **0.0** |
| ^2^**RC** | -2029.389704 | **0.0** | -2028.892609 | **-0.1** | -2029.861436 | **-0.1** | -2029.364341 | **-0.2** | -2029.885132 | **0.0** | -2029.388037 | **0.0** | -2028.963826 | **0.1** |
| ^4^**TS** | -2029.365640 | **15.1** | -2028.874257 | **11.4** | -2029.845535 | **9.9** | -2029.354152 | **6.2** | -2029.868226 | **10.6** | -2029.376843 | **7.0** | -2028.941406 | **14.2** |
| ^2^**TS** | -2029.369273 | **12.8** | -2028.876838 | **9.8** | -2029.847519 | **8.6** | -2029.355084 | **5.7** | -2029.871248 | **8.7** | -2029.378813 | **5.8** | -2028.942955 | **13.2** |
| ^4^**PC**_reb_ | -2029.470992 | **-51.1** | -2028.972978 | **-50.5** | -2029.953434 | **-57.8** | -2029.455420 | **-57.3** | -2029.976845 | **-57.5** | -2029.478831 | **-57.0** | -2029.042322 | **-49.1** |
| ^2^**PC**_reb_ | -2029.478124 | **-55.5** | -2028.978010 | **-53.7** | -2029.956611 | **-59.8** | -2029.456497 | **-58.0** | -2029.975275 | **-56.5** | -2029.475161 | **-54.7** | -2029.042386 | **-49.2** |
| ^4^**PC**_DHA_ | -2029.447573 | **-36.4** | -2028.953642 | **-38.4** | -2029.936284 | **-47.1** | -2029.442353 | **-49.1** | -2029.963257 | **-49.0** | -2029.469326 | **-51.0** | -2029.025552 | **-38.6** |
| ^2^**PC**_DHA_ | -2029.459388 | **-43.8** | -2028.963417 | **-44.5** | -2029.943448 | **-51.6** | -2029.447477 | **-52.3** | -2029.965645 | **-50.5** | -2029.469674 | **-51.3** | -2029.031653 | **-42.4** |

*^a^* Absolute SCF energies ***E*** are in a.u. and relative SCF energies **Δ*E*** are in kcal/mol.

**Supplementary Table 8.** The Mulliken spin density and charge of various reaction species for C-H hydroxylation and dual hydrogen abstraction on the cyclopropyl group of BCA calculated at the B3LYP/B1 level.

|  | Spin density | | | | | |  | Charge | | | | | |
| --- | --- | --- | --- | --- | --- | --- | --- | --- | --- | --- | --- | --- | --- |
|  | Fe | O | por | SH | H | rest |  | Fe | O | por | SH | H | rest |
| ^4^**RC** | 1.10 | 0.92 | 0.45 | 0.53 | 0.00 | 0.00 |  | 0.48 | -0.40 | -0.03 | -0.05 | 0.18 | -0.18 |
| ^2^**RC** | 1.21 | 0.88 | -0.52 | -0.57 | 0.00 | 0.00 |  | 0.49 | -0.40 | -0.04 | -0.05 | 0.18 | -0.18 |
| ^4^**TS** | 1.38 | 0.68 | 0.02 | 0.31 | -0.01 | 0.62 |  | 0.41 | -0.58 | -0.27 | 0.00 | 0.39 | 0.06 |
| ^2^**TS** | 1.58 | 0.16 | -0.21 | -0.10 | 0.00 | -0.43 |  | 0.40 | -0.54 | -0.23 | -0.05 | 0.34 | 0.07 |
| ^4^**PC**_reb_ | 2.52 | 0.01 | 0.01 | 0.47 | 0.00 | 0.00 |  | 0.45 | -0.61 | -0.40 | -0.15 | 0.42 | 0.29 |
| ^2^**PC**_reb_ | 1.08 | 0.00 | -0.08 | 0.00 | 0.00 | 0.00 |  | 0.23 | -0.61 | -0.43 | 0.04 | 0.43 | 0.34 |
| ^4^**PC**_DHA_ | 2.54 | 0.02 | -0.02 | 0.45 | 0.00 | 0.00 |  | 0.44 | -0.78 | -0.42 | -0.18 | 0.42 | 0.52 |
| ^2^**PC**_DHA_ | 1.05 | 0.00 | -0.08 | 0.03 | 0.00 | 0.00 |  | 0.22 | -0.77 | -0.45 | 0.01 | 0.44 | 0.56 |

**Supplementary Table 9.** Computational deuterium kinetic isotope effects (KIEs) for three reaction pathways.

|  | Methylene | Cyclopropyl | N-H activation |
| --- | --- | --- | --- |
| KIE_HS_ | 5.9 | 6.6 | 5.9 |
| KIE_LS_ | 4.5 | 4.1 | 6.1 |

## Supplementary Cartesian Coordinates

- 1. **Methylene Oxidation.**

^4^**RC**

26 6.984219000 -13.604778000 21.799068000

7 5.813133000 -12.818397000 23.243355000

6 6.153696000 -12.618247000 24.556912000

6 5.010776000 -12.158121000 25.307802000

6 3.970883000 -12.089045000 24.432143000

6 4.483959000 -12.504480000 23.149106000

1 5.021109000 -11.923159000 26.364407000

1 2.948719000 -11.785648000 24.618198000

6 3.727367000 -12.562995000 21.982701000

1 2.678687000 -12.292854000 22.055917000

7 5.491025000 -13.341665000 20.465583000

6 4.199385000 -12.944840000 20.737648000

6 3.396261000 -13.005881000 19.541649000

6 4.205857000 -13.459964000 18.545608000

6 5.507560000 -13.674260000 19.129529000

1 2.348955000 -12.737494000 19.490391000

1 3.962680000 -13.635873000 17.505591000

6 6.607471000 -14.160371000 18.440362000

1 6.473189000 -14.392090000 17.388385000

7 8.217524000 -14.121956000 20.287287000

6 7.872677000 -14.353017000 18.980651000

6 9.017052000 -14.814924000 18.232272000

6 10.061243000 -14.859798000 19.102522000

6 9.552211000 -14.432526000 20.383509000

1 9.005126000 -15.060431000 17.178142000

1 11.086148000 -15.152245000 18.913598000

6 10.308322000 -14.359536000 21.543554000

1 11.355597000 -14.637294000 21.472913000

7 8.553753000 -13.581171000 23.064647000

6 9.836328000 -13.967848000 22.792327000

6 10.647442000 -13.904455000 23.984782000

6 9.832663000 -13.473416000 24.985651000

6 8.528990000 -13.274803000 24.398545000

1 11.698754000 -14.158076000 24.030191000

1 10.074001000 -13.298115000 26.026167000

6 7.416009000 -12.823890000 25.097354000

1 7.543131000 -12.618528000 26.155983000

8 6.583821000 -15.139549000 22.168276000

16 7.518164000 -11.247461000 20.946852000

1 6.385200000 -10.634499000 21.358709000

1 -0.470643000 -17.108812000 22.316491000

6 0.576217000 -16.869905000 22.489122000

6 0.956704000 -16.195357000 23.653182000

6 1.536497000 -17.241145000 21.547967000

6 2.302498000 -15.890840000 23.863629000

1 0.208230000 -15.908727000 24.388065000

6 2.891753000 -16.944777000 21.754168000

1 1.245898000 -17.754718000 20.635911000

6 3.263500000 -16.259583000 22.917731000

1 2.608786000 -15.358439000 24.761000000

6 3.940128000 -17.376926000 20.747587000

1 4.309732000 -16.007952000 23.074550000

7 3.518620000 -17.089222000 19.369592000

1 4.906153000 -16.911934000 21.006625000

1 4.089151000 -18.464191000 20.812436000

6 4.463287000 -17.536621000 18.379936000

1 3.372287000 -16.084522000 19.272572000

6 4.298915000 -18.939147000 17.849485000

6 3.949071000 -17.753669000 16.980009000

1 5.495036000 -17.177559000 18.471310000

1 5.191330000 -19.512533000 17.613594000

1 3.474326000 -19.518776000 18.253991000

1 2.894753000 -17.554616000 16.811294000

1 4.600250000 -17.511684000 16.143923000

^2^**RC**

26 6.921221000 -13.594209000 21.755426000

7 5.764817000 -12.808034000 23.208891000

6 6.111844000 -12.592851000 24.515723000

6 4.970279000 -12.138692000 25.272482000

6 3.922940000 -12.086884000 24.404472000

6 4.428624000 -12.509597000 23.121326000

1 4.986825000 -11.894749000 26.326986000

1 2.899542000 -11.791370000 24.596331000

6 3.662506000 -12.598492000 21.967606000

1 2.612212000 -12.336247000 22.045764000

7 5.412902000 -13.380990000 20.441321000

6 4.126649000 -13.003201000 20.722004000

6 3.311965000 -13.067120000 19.533445000

6 4.126954000 -13.486886000 18.525519000

6 5.434057000 -13.679832000 19.106736000

1 2.259791000 -12.816894000 19.492612000

1 3.884999000 -13.648961000 17.482958000

6 6.551535000 -14.108135000 18.395918000

1 6.419629000 -14.308448000 17.337070000

7 8.159457000 -14.107511000 20.243785000

6 7.814689000 -14.311746000 18.930032000

6 8.953886000 -14.783417000 18.179966000

6 9.988647000 -14.874042000 19.057919000

6 9.482942000 -14.449197000 20.341414000

1 8.943779000 -15.010572000 17.121679000

1 11.007104000 -15.189633000 18.871491000

6 10.245536000 -14.384993000 21.501373000

1 11.283573000 -14.695415000 21.432544000

7 8.514246000 -13.513805000 23.003152000

6 9.796784000 -13.934660000 22.731907000

6 10.610975000 -13.851376000 23.919937000

6 9.809079000 -13.391352000 24.917160000

6 8.499671000 -13.196347000 24.343771000

1 11.659338000 -14.116890000 23.965800000

1 10.061235000 -13.201924000 25.952685000

6 7.385583000 -12.771832000 25.048586000

1 7.515398000 -12.562725000 26.106131000

8 6.559576000 -15.132788000 22.139871000

16 7.858694000 -11.249003000 21.251627000

1 7.633610000 -10.697304000 22.465822000

1 -0.505474000 -17.086042000 22.196350000

6 0.537059000 -16.849705000 22.396532000

6 0.886964000 -16.165003000 23.564202000

6 1.522269000 -17.234292000 21.486937000

6 2.227561000 -15.863835000 23.810000000

1 0.119057000 -15.867839000 24.274445000

6 2.872293000 -16.940913000 21.728520000

1 1.255914000 -17.755764000 20.572018000

6 3.213479000 -16.245711000 22.895503000

1 2.510367000 -15.323763000 24.710502000

6 3.947631000 -17.385730000 20.756370000

1 4.255765000 -15.995745000 23.078697000

7 3.559659000 -17.123855000 19.363409000

1 4.904477000 -16.911354000 21.031265000

1 4.099352000 -18.471050000 20.844096000

6 4.531638000 -17.581707000 18.405473000

1 3.410062000 -16.121840000 19.245880000

6 4.391407000 -18.995579000 17.898907000

6 4.052459000 -17.830206000 16.998397000

1 5.557936000 -17.211825000 18.514084000

1 5.293751000 -19.565787000 17.695285000

1 3.562254000 -19.574326000 18.295288000

1 3.000796000 -17.643438000 16.801526000

1 4.721163000 -17.598898000 16.173206000

^4^**TS**

26 6.690283000 -13.675531000 21.694911000

7 5.775505000 -12.946559000 23.329034000

6 6.310604000 -12.846852000 24.585638000

6 5.320718000 -12.348066000 25.510046000

6 4.175813000 -12.164766000 24.797259000

6 4.465893000 -12.545087000 23.435228000

1 5.495462000 -12.174044000 26.564214000

1 3.214618000 -11.806538000 25.143629000

6 3.541071000 -12.500405000 22.400289000

1 2.539688000 -12.159393000 22.643082000

7 4.992718000 -13.301400000 20.600166000

6 3.796102000 -12.840670000 21.077559000

6 2.842320000 -12.720891000 19.999506000

6 3.486587000 -13.103729000 18.863074000

6 4.832888000 -13.462284000 19.249905000

1 1.820949000 -12.382059000 20.115504000

1 3.106205000 -13.136371000 17.849719000

6 5.816825000 -13.903590000 18.371492000

1 5.551418000 -13.977823000 17.321024000

7 7.643243000 -14.208477000 19.991022000

6 7.117318000 -14.242800000 18.722341000

6 8.110120000 -14.722983000 17.790962000

6 9.234263000 -14.985079000 18.513656000

6 8.930527000 -14.661027000 19.886877000

1 7.949941000 -14.838358000 16.726419000

1 10.188916000 -15.358738000 18.165518000

6 9.823165000 -14.793655000 20.945059000

1 10.816653000 -15.167045000 20.716640000

7 8.362452000 -13.970480000 22.736471000

6 9.551465000 -14.467529000 22.264474000

6 10.511392000 -14.563596000 23.340437000

6 9.892873000 -14.105338000 24.460629000

6 8.551337000 -13.732876000 24.073630000

1 11.524913000 -14.927492000 23.228946000

1 10.290576000 -14.015265000 25.463504000

6 7.606620000 -13.202346000 24.938559000

1 7.897469000 -13.066856000 25.975684000

8 6.162313000 -15.267698000 22.018964000

16 7.308880000 -11.465698000 21.043694000

1 6.412570000 -10.788758000 21.795935000

1 -0.269291000 -16.068291000 20.687629000

6 0.607925000 -16.073976000 21.329876000

6 0.487410000 -15.744368000 22.681141000

6 1.849010000 -16.428755000 20.798921000

6 1.620868000 -15.768895000 23.499826000

1 -0.480938000 -15.474767000 23.094514000

6 2.992500000 -16.456355000 21.612052000

1 1.930124000 -16.717389000 19.755383000

6 2.861482000 -16.115203000 22.969902000

1 1.538916000 -15.511887000 24.552744000

6 4.338981000 -16.842973000 21.089317000

1 3.745336000 -16.111041000 23.602564000

7 4.458292000 -17.000519000 19.688490000

1 5.165590000 -15.964549000 21.484786000

1 4.757054000 -17.716869000 21.602519000

6 5.671166000 -17.593477000 19.189374000

1 4.189291000 -16.165395000 19.171961000

6 5.750861000 -19.098552000 19.140253000

6 5.608648000 -18.302063000 17.863776000

1 6.590909000 -17.056319000 19.427354000

1 6.705758000 -19.565916000 19.362434000

1 4.880241000 -19.651209000 19.481104000

1 4.645083000 -18.335197000 17.362975000

1 6.464762000 -18.215369000 17.200958000

^2^**TS**

26 6.589979000 -13.673151000 21.637316000

7 5.724817000 -12.907299000 23.296883000

6 6.267001000 -12.845468000 24.555123000

6 5.298347000 -12.316568000 25.486038000

6 4.165208000 -12.067986000 24.774346000

6 4.441218000 -12.447191000 23.408922000

1 5.480375000 -12.163066000 26.542194000

1 3.222012000 -11.667111000 25.123215000

6 3.520455000 -12.368212000 22.371513000

1 2.536874000 -11.973810000 22.605826000

7 4.924071000 -13.289520000 20.578886000

6 3.756383000 -12.749804000 21.060105000

6 2.793305000 -12.616926000 19.994460000

6 3.395471000 -13.068833000 18.861291000

6 4.729226000 -13.477456000 19.234410000

1 1.793217000 -12.221495000 20.116564000

1 2.996116000 -13.114414000 17.855885000

6 5.677896000 -13.965138000 18.343245000

1 5.388394000 -14.054749000 17.300579000

7 7.520691000 -14.230130000 19.936944000

6 6.985020000 -14.298595000 18.677672000

6 7.974951000 -14.771923000 17.740498000

6 9.115644000 -14.987991000 18.451832000

6 8.824283000 -14.646436000 19.823271000

1 7.803589000 -14.908143000 16.680225000

1 10.076130000 -15.339559000 18.096901000

6 9.740488000 -14.742236000 20.861172000

1 10.734609000 -15.101985000 20.614132000

7 8.313353000 -13.924319000 22.676921000

6 9.494412000 -14.411024000 22.185838000

6 10.464694000 -14.527188000 23.250982000

6 9.849765000 -14.106883000 24.388442000

6 8.503575000 -13.731627000 24.019176000

1 11.478837000 -14.883723000 23.123021000

1 10.252461000 -14.045107000 25.391482000

6 7.556603000 -13.228512000 24.899726000

1 7.847813000 -13.121290000 25.940109000

8 6.196352000 -15.235822000 22.112258000

16 7.425831000 -11.463898000 21.122775000

1 7.481104000 -10.988980000 22.386864000

1 -0.242444000 -16.338710000 20.636461000

6 0.626754000 -16.241770000 21.282218000

6 0.477171000 -15.784557000 22.592779000

6 1.888486000 -16.590845000 20.798084000

6 1.600889000 -15.673805000 23.417052000

1 -0.506735000 -15.518486000 22.970157000

6 3.021168000 -16.484215000 21.618604000

1 1.998322000 -16.974163000 19.788089000

6 2.862389000 -16.013737000 22.932848000

1 1.495967000 -15.314028000 24.437279000

6 4.390773000 -16.875964000 21.143106000

1 3.739168000 -15.903035000 23.565360000

7 4.538382000 -17.039832000 19.743232000

1 5.168275000 -16.035954000 21.543314000

1 4.771220000 -17.766794000 21.658943000

6 5.758626000 -17.637338000 19.271051000

1 4.279719000 -16.206423000 19.217064000

6 5.833670000 -19.142631000 19.214232000

6 5.726856000 -18.336720000 17.939946000

1 6.674281000 -17.104889000 19.535824000

1 6.780785000 -19.615303000 19.457366000

1 4.952521000 -19.694235000 19.528819000

1 4.776161000 -18.363057000 17.414670000

1 6.599742000 -18.247754000 17.299855000

^4^**PC**_reb_

26 6.710501000 -13.187096000 21.363002000

7 5.923171000 -12.870106000 23.182179000

6 6.457290000 -13.247074000 24.393339000

6 5.650902000 -12.740948000 25.472870000

6 4.613478000 -12.063217000 24.906102000

6 4.784054000 -12.156850000 23.479720000

1 5.858704000 -12.900333000 26.523265000

1 3.794560000 -11.551814000 25.395807000

6 3.898010000 -11.624080000 22.553424000

1 3.044079000 -11.073650000 22.935554000

7 5.033503000 -12.409892000 20.523848000

6 4.019578000 -11.747181000 21.177616000

6 3.100970000 -11.174965000 20.226366000

6 3.575725000 -11.480337000 18.987207000

6 4.785395000 -12.239193000 19.179958000

1 2.215935000 -10.609418000 20.488798000

1 3.161317000 -11.217319000 18.022300000

6 5.587767000 -12.708621000 18.151018000

1 5.275276000 -12.498797000 17.132985000

7 7.312785000 -13.779021000 19.539242000

6 6.764009000 -13.424057000 18.326816000

6 7.573710000 -13.925049000 17.247902000

6 8.618621000 -14.591663000 17.814641000

6 8.447848000 -14.499805000 19.240416000

1 7.356437000 -13.780618000 16.197220000

1 9.434753000 -15.106991000 17.324445000

6 9.296967000 -15.087519000 20.167443000

1 10.154452000 -15.633490000 19.787033000

7 8.111778000 -14.377701000 22.195531000

6 9.126700000 -15.034495000 21.542190000

6 10.013754000 -15.658407000 22.493051000

6 9.532533000 -15.362503000 23.731016000

6 8.352082000 -14.557306000 23.536505000

1 10.889373000 -16.237971000 22.229566000

1 9.929266000 -15.649965000 24.696351000

6 7.590541000 -14.028682000 24.567076000

1 7.898250000 -14.247739000 25.584587000

8 4.824438000 -15.374224000 21.336977000

16 8.059126000 -11.162824000 21.267874000

1 7.721303000 -10.712623000 22.497602000

1 0.079860000 -18.735473000 21.220792000

6 0.654593000 -17.850362000 21.481717000

6 0.009237000 -16.722567000 21.992959000

6 2.038925000 -17.848454000 21.303436000

6 0.759454000 -15.594297000 22.328686000

1 -1.068818000 -16.724478000 22.131549000

6 2.798360000 -16.715531000 21.628664000

1 2.544104000 -18.724241000 20.909860000

6 2.144109000 -15.590751000 22.150561000

1 0.267257000 -14.713128000 22.732539000

6 4.315932000 -16.715525000 21.462731000

1 2.725372000 -14.714613000 22.419912000

7 4.719011000 -17.611034000 20.404572000

1 4.326632000 -14.907494000 20.644058000

1 4.797438000 -17.070837000 22.380463000

6 6.144216000 -17.766170000 20.240151000

1 4.273062000 -17.351199000 19.525202000

6 6.824209000 -18.818167000 21.081255000

6 6.628698000 -19.039896000 19.599930000

1 6.720860000 -16.862835000 20.041480000

1 7.822543000 -18.605599000 21.452952000

1 6.196085000 -19.392641000 21.755880000

1 5.872764000 -19.760463000 19.300918000

1 7.493647000 -18.987528000 18.944265000

^2^**PC**_reb_

26 6.400260000 -13.396058000 21.245927000

7 5.529361000 -13.156822000 23.042438000

6 5.913620000 -13.698269000 24.244951000

6 5.067751000 -13.209209000 25.307168000

6 4.178996000 -12.351131000 24.736012000

6 4.471348000 -12.326691000 23.321948000

1 5.160909000 -13.490250000 26.348550000

1 3.387750000 -11.784966000 25.211141000

6 3.744623000 -11.599310000 22.386718000

1 2.931801000 -10.983134000 22.759295000

7 4.926942000 -12.333418000 20.361609000

6 3.953864000 -11.612999000 21.013347000

6 3.152383000 -10.883449000 20.060330000

6 3.653352000 -11.164409000 18.825757000

6 4.770387000 -12.055613000 19.023268000

1 2.317430000 -10.244431000 20.318961000

1 3.317726000 -10.800774000 17.862762000

6 5.592939000 -12.527724000 18.008468000

1 5.356238000 -12.232018000 16.990996000

7 7.180447000 -13.751055000 19.422028000

6 6.729072000 -13.302332000 18.205926000

6 7.629436000 -13.713586000 17.154347000

6 8.641647000 -14.398489000 17.752313000

6 8.352878000 -14.417835000 19.167870000

1 7.494098000 -13.488726000 16.103938000

1 9.508836000 -14.857124000 17.294026000

6 9.143823000 -15.050943000 20.117862000

1 10.050957000 -15.534375000 19.767712000

7 7.760589000 -14.600094000 22.097782000

6 8.857635000 -15.141705000 21.474270000

6 9.668362000 -15.861144000 22.428646000

6 9.046057000 -15.753567000 23.634978000

6 7.864495000 -14.953130000 23.422259000

1 10.589639000 -16.377397000 22.189147000

1 9.350879000 -16.161010000 24.590647000

6 6.990406000 -14.556767000 24.426772000

1 7.189767000 -14.907531000 25.434721000

8 5.040847000 -15.150244000 20.842611000

16 7.671905000 -11.571492000 21.494380000

1 8.109247000 -11.816487000 22.750752000

1 0.606474000 -18.638295000 22.266106000

6 1.140475000 -17.692250000 22.228712000

6 0.486314000 -16.504666000 22.562571000

6 2.481922000 -17.672399000 21.845273000

6 1.186497000 -15.298596000 22.515464000

1 -0.558840000 -16.520156000 22.860361000

6 3.189538000 -16.462597000 21.785376000

1 2.993209000 -18.593672000 21.587975000

6 2.529160000 -15.276412000 22.133043000

1 0.690555000 -14.368165000 22.779433000

6 4.662413000 -16.453198000 21.386305000

1 3.066081000 -14.335287000 22.112446000

7 4.961106000 -17.548842000 20.511663000

1 4.359984000 -14.830271000 20.226538000

1 5.311696000 -16.539862000 22.262196000

6 6.351362000 -17.767247000 20.196871000

1 4.382338000 -17.518727000 19.673882000

6 7.143706000 -18.632684000 21.145439000

6 6.747385000 -19.161850000 19.788172000

1 6.887740000 -16.943099000 19.727574000

1 8.181069000 -18.369547000 21.327539000

1 6.614785000 -19.030366000 22.006771000

1 5.960132000 -19.909810000 19.756013000

1 7.512686000 -19.273127000 19.025014000

^4^**PC**_DHA_

26 6.455204000 -13.015799000 21.414275000

7 6.225685000 -12.287326000 23.296805000

6 7.149176000 -12.324710000 24.313721000

6 6.674383000 -11.565968000 25.445188000

6 5.461550000 -11.055529000 25.094852000

6 5.193015000 -11.502645000 23.749828000

1 7.214749000 -11.443176000 26.375389000

1 4.800685000 -10.427051000 25.678400000

6 4.059934000 -11.165525000 23.023543000

1 3.320813000 -10.537648000 23.511251000

7 4.614687000 -12.351052000 20.943324000

6 3.796263000 -11.569710000 21.720785000

6 2.592344000 -11.242718000 20.998960000

6 2.688497000 -11.844032000 19.779305000

6 3.951697000 -12.538057000 19.755946000

1 1.786830000 -10.633197000 21.388685000

1 1.979372000 -11.827423000 18.961278000

6 4.402018000 -13.306756000 18.690342000

1 3.768502000 -13.362838000 17.810817000

7 6.520791000 -14.051943000 19.679897000

6 5.598699000 -14.007741000 18.663525000

6 6.063209000 -14.779132000 17.536125000

6 7.284453000 -15.275397000 17.875659000

6 7.565214000 -14.810081000 19.212989000

1 5.516940000 -14.905726000 16.609998000

1 7.950096000 -15.892463000 17.285207000

6 8.734735000 -15.084140000 19.908968000

1 9.483816000 -15.693107000 19.412306000

7 8.199055000 -13.855709000 21.970614000

6 9.019257000 -14.641529000 21.194799000

6 10.220895000 -14.967538000 21.918150000

6 10.116562000 -14.379295000 23.144079000

6 8.852598000 -13.690243000 23.170650000

1 11.030482000 -15.571371000 21.527725000

1 10.822336000 -14.403152000 23.964865000

6 8.371800000 -12.980208000 24.263975000

1 9.004758000 -12.929184000 25.144472000

8 5.235128000 -14.930690000 22.416055000

16 7.454075000 -10.969577000 20.498419000

1 8.741588000 -11.356032000 20.647660000

1 1.157596000 -14.726359000 20.696472000

6 1.362300000 -15.780242000 20.528846000

6 0.426673000 -16.581838000 19.865607000

6 2.565717000 -16.320133000 20.974492000

6 0.697153000 -17.934253000 19.647928000

1 -0.510530000 -16.152871000 19.520614000

6 2.848892000 -17.681221000 20.755565000

1 3.282494000 -15.691921000 21.492694000

6 1.901522000 -18.478301000 20.090642000

1 -0.026256000 -18.562032000 19.134992000

6 4.099086000 -18.320027000 21.190821000

1 2.115181000 -19.531453000 19.920521000

7 5.063434000 -17.723943000 21.785088000

1 5.427244000 -14.881639000 23.364602000

1 4.166537000 -19.394078000 20.970952000

6 6.236377000 -18.473505000 22.160288000

1 5.278584000 -15.889140000 22.169839000

6 6.748353000 -19.696645000 21.435660000

6 7.484527000 -18.376404000 21.324005000

1 6.393447000 -18.444301000 23.237847000

1 7.186871000 -20.494918000 22.028541000

1 6.222858000 -20.043958000 20.550837000

1 7.400642000 -17.838841000 20.383776000

1 8.436629000 -18.250009000 21.831559000

^2^**PC**_DHA_

26 6.304767000 -13.079583000 21.434122000

7 6.228032000 -12.377132000 23.333523000

6 7.192456000 -12.553816000 24.297195000

6 6.846483000 -11.814385000 25.487421000

6 5.670138000 -11.178555000 25.229133000

6 5.295929000 -11.526706000 23.880119000

1 7.439953000 -11.791365000 26.392845000

1 5.099749000 -10.526024000 25.878207000

6 4.164179000 -11.055370000 23.227026000

1 3.505373000 -10.392136000 23.779193000

7 4.545545000 -12.158090000 21.070757000

6 3.822799000 -11.354401000 21.914005000

6 2.648047000 -10.850534000 21.240442000

6 2.673552000 -11.359102000 19.978854000

6 3.864001000 -12.174240000 19.882280000

1 1.913626000 -10.193935000 21.689767000

1 1.965062000 -11.204981000 19.174489000

6 4.235953000 -12.878344000 18.743849000

1 3.593144000 -12.789884000 17.873054000

7 6.290456000 -13.915176000 19.603934000

6 5.363153000 -13.681047000 18.620347000

6 5.721718000 -14.396075000 17.417460000

6 6.881753000 -15.056280000 17.682968000

6 7.232132000 -14.746047000 19.048879000

1 5.149335000 -14.381264000 16.498481000

1 7.460401000 -15.694877000 17.027296000

6 8.371635000 -15.208379000 19.695357000

1 9.045797000 -15.846458000 19.131717000

7 7.993002000 -14.101285000 21.850629000

6 8.723955000 -14.896126000 21.003065000

6 9.924515000 -15.355097000 21.660160000

6 9.911161000 -14.824835000 22.914717000

6 8.704266000 -14.039256000 23.023400000

1 10.672086000 -15.992566000 21.204592000

1 10.645239000 -14.938134000 23.702768000

6 8.336662000 -13.329636000 24.160641000

1 9.008986000 -13.375894000 25.012226000

8 5.113396000 -14.689079000 22.165770000

16 7.449398000 -11.278044000 20.741497000

1 8.335388000 -11.912378000 19.940425000

1 1.449699000 -14.953256000 19.652473000

6 1.671517000 -16.009651000 19.775739000

6 0.822754000 -16.976660000 19.226539000

6 2.809216000 -16.386899000 20.483640000

6 1.114468000 -18.332351000 19.389417000

1 -0.063155000 -16.673630000 18.674676000

6 3.113217000 -17.750709000 20.651393000

1 3.452698000 -15.626965000 20.911958000

6 2.252199000 -18.714220000 20.097654000

1 0.458876000 -19.088671000 18.966798000

6 4.292887000 -18.233479000 21.382525000

1 2.481092000 -19.770191000 20.224847000

7 5.189915000 -17.497271000 21.924546000

1 5.057995000 -14.541646000 23.123535000

1 4.369467000 -19.326534000 21.447905000

6 6.301083000 -18.111365000 22.607354000

1 5.291874000 -15.660141000 22.022498000

6 6.818175000 -19.508485000 22.352960000

6 7.610212000 -18.304828000 21.887257000

1 6.384675000 -17.743210000 23.628768000

1 7.177926000 -20.075915000 23.207107000

1 6.351159000 -20.119815000 21.586526000

1 7.610489000 -18.094393000 20.821534000

1. 8.525564000 -18.026652000 22.400761000
   1. **Cyclopropyl Oxidation.**

^4^**RC**

26 6.497924000 -13.553977000 21.630826000

7 5.506405000 -12.742402000 23.190430000

6 6.028293000 -12.422031000 24.417610000

6 4.981719000 -11.987573000 25.310872000

6 3.816644000 -12.058920000 24.610607000

6 4.157446000 -12.531452000 23.290527000

1 5.136512000 -11.673804000 26.335260000

1 2.813641000 -11.817230000 24.937491000

6 3.236266000 -12.728452000 22.265987000

1 2.192148000 -12.538579000 22.492314000

7 4.804382000 -13.478741000 20.534056000

6 3.541856000 -13.157869000 20.984719000

6 2.571525000 -13.359837000 19.936649000

6 3.253646000 -13.819365000 18.851855000

6 4.642395000 -13.899507000 19.232708000

1 1.509647000 -13.178222000 20.040164000

1 2.867550000 -14.087227000 17.876800000

6 5.658099000 -14.359015000 18.409614000

1 5.383839000 -14.671670000 17.406894000

7 7.522585000 -14.096127000 19.978433000

6 7.000975000 -14.434965000 18.756694000

6 8.048491000 -14.876502000 17.867384000

6 9.212880000 -14.799907000 18.566053000

6 8.874773000 -14.318265000 19.883786000

1 7.894116000 -15.193238000 16.843926000

1 10.215361000 -15.041986000 18.237277000

6 9.790807000 -14.119387000 20.905736000

1 10.832383000 -14.336875000 20.689420000

7 8.236284000 -13.342572000 22.631558000

6 9.487010000 -13.668797000 22.186544000

6 10.463849000 -13.466350000 23.230023000

6 9.782413000 -13.010663000 24.316001000

6 8.393481000 -12.937545000 23.929592000

1 11.525374000 -13.650719000 23.125953000

1 10.166481000 -12.742109000 25.291720000

6 7.370418000 -12.504606000 24.763928000

1 7.642044000 -12.211659000 25.773597000

8 6.265877000 -15.080673000 22.147855000

16 6.717763000 -11.229697000 20.573198000

1 5.634569000 -10.659523000 21.148363000

1 0.421813000 -14.217219000 25.548772000

6 0.427217000 -14.825602000 24.647278000

6 -0.750091000 -15.007194000 23.917927000

6 1.615492000 -15.422163000 24.219956000

6 -0.727480000 -15.792988000 22.763212000

1 -1.675802000 -14.541448000 24.246414000

6 1.647718000 -16.217569000 23.066211000

1 2.531491000 -15.267599000 24.786145000

6 0.460207000 -16.395043000 22.342897000

1 -1.639188000 -15.941315000 22.189261000

6 2.938090000 -16.891087000 22.637590000

1 0.485497000 -17.005015000 21.445113000

7 3.023994000 -17.049780000 21.183213000

1 3.796666000 -16.337498000 23.055656000

1 2.984084000 -17.900285000 23.072240000

6 4.237856000 -17.708188000 20.763288000

1 2.977135000 -16.128470000 20.748252000

6 4.210712000 -19.213811000 20.676458000

6 4.226229000 -18.380028000 19.415601000

1 5.180241000 -17.252231000 21.080275000

1 5.107142000 -19.764098000 20.949136000

1 3.275418000 -19.702173000 20.935122000

1 3.302315000 -18.323214000 18.847044000

1 5.134712000 -18.352683000 18.819755000

^2^**RC**

26 6.500356000 -13.549873000 21.638281000

7 5.505477000 -12.741162000 23.194711000

6 6.024024000 -12.425232000 24.424262000

6 4.975875000 -11.991533000 25.315734000

6 3.812826000 -12.058515000 24.611670000

6 4.156077000 -12.528806000 23.291490000

1 5.128264000 -11.680790000 26.341406000

1 2.809291000 -11.816059000 24.936341000

6 3.234967000 -12.725997000 22.266951000

1 2.190811000 -12.537756000 22.494471000

7 4.802900000 -13.472057000 20.534650000

6 3.539487000 -13.158393000 20.986351000

6 2.568272000 -13.371455000 19.941080000

6 3.251349000 -13.833127000 18.857756000

6 4.641409000 -13.902728000 19.236874000

1 1.505392000 -13.196410000 20.045623000

1 2.865056000 -14.109289000 17.885079000

6 5.659307000 -14.359783000 18.414954000

1 5.386164000 -14.677752000 17.413588000

7 7.525271000 -14.085624000 19.981680000

6 7.002552000 -14.429983000 18.761816000

6 8.050416000 -14.871599000 17.872708000

6 9.215423000 -14.790211000 18.569749000

6 8.877163000 -14.305865000 19.886476000

1 7.895657000 -15.191907000 16.850406000

1 10.218074000 -15.031232000 18.240706000

6 9.792417000 -14.102895000 20.908442000

1 10.834966000 -14.316191000 20.692680000

7 8.233200000 -13.337141000 22.636651000

6 9.486216000 -13.654163000 22.189154000

6 10.462495000 -13.447424000 23.231823000

6 9.779385000 -12.999735000 24.320162000

6 8.389817000 -12.934675000 23.936120000

1 11.525192000 -13.623800000 23.125816000

1 10.163107000 -12.730892000 25.295941000

6 7.365553000 -12.509201000 24.772600000

1 7.636038000 -12.220058000 25.783651000

8 6.243075000 -15.081725000 22.120880000

16 6.698426000 -11.220134000 20.561788000

1 5.618138000 -10.653217000 21.145536000

1 0.451756000 -14.217416000 25.554091000

6 0.452083000 -14.819918000 24.648629000

6 -0.725402000 -14.982178000 23.914957000

6 1.634081000 -15.428282000 24.220594000

6 -0.709246000 -15.760781000 22.755284000

1 -1.646214000 -14.507203000 24.244018000

6 1.659745000 -16.216621000 23.061848000

1 2.550381000 -15.288401000 24.790094000

6 0.472101000 -16.374833000 22.334344000

1 -1.621137000 -15.894259000 22.177968000

6 2.943103000 -16.903054000 22.632441000

1 0.492419000 -16.979451000 21.432824000

7 3.028382000 -17.058985000 21.177743000

1 3.807226000 -16.359800000 23.052389000

1 2.977434000 -17.913834000 23.064472000

6 4.233027000 -17.733587000 20.757084000

1 2.995705000 -16.135799000 20.745522000

6 4.185302000 -19.238676000 20.670242000

6 4.211418000 -18.405227000 19.409367000

1 5.181542000 -17.290098000 21.073151000

1 5.074289000 -19.801198000 20.942352000

1 3.243642000 -19.714361000 20.929480000

1 3.288009000 -18.335842000 18.841364000

1 5.119807000 -18.390546000 18.812924000

^4^**TS**

26 6.557121000 -13.624168000 21.459159000

7 5.566020000 -12.868323000 23.044362000

6 6.084496000 -12.632595000 24.288979000

6 5.050859000 -12.159132000 25.177376000

6 3.896373000 -12.127850000 24.455799000

6 4.223059000 -12.581130000 23.124583000

1 5.205769000 -11.894427000 26.215684000

1 2.907297000 -11.826055000 24.776917000

6 3.298548000 -12.712994000 22.097648000

1 2.269907000 -12.443487000 22.316492000

7 4.819686000 -13.537779000 20.363074000

6 3.585364000 -13.154234000 20.809708000

6 2.618236000 -13.249530000 19.738806000

6 3.296636000 -13.682373000 18.638671000

6 4.673969000 -13.851074000 19.038426000

1 1.569511000 -12.995172000 19.829161000

1 2.917696000 -13.861505000 17.640388000

6 5.696894000 -14.259757000 18.191315000

1 5.434823000 -14.473808000 17.159683000

7 7.556191000 -14.194103000 19.802215000

6 7.028119000 -14.414209000 18.553519000

6 8.063951000 -14.869280000 17.656250000

6 9.214552000 -14.930016000 18.380596000

6 8.884253000 -14.511637000 19.722063000

1 7.910383000 -15.108673000 16.611580000

1 10.202520000 -15.230099000 18.055219000

6 9.788064000 -14.461769000 20.776829000

1 10.815540000 -14.743838000 20.569503000

7 8.243498000 -13.676418000 22.514218000

6 9.479439000 -14.073659000 22.070982000

6 10.440479000 -13.996576000 23.147636000

6 9.773162000 -13.536334000 24.238465000

6 8.400973000 -13.332582000 23.833495000

1 11.487037000 -14.258144000 23.056153000

1 10.155701000 -13.341447000 25.232315000

6 7.407233000 -12.841100000 24.663838000

1 7.679669000 -12.607885000 25.688409000

8 6.193610000 -15.250091000 21.905302000

16 6.939986000 -11.445028000 20.637714000

1 6.082879000 -10.780608000 21.444818000

1 -0.603174000 -14.248834000 25.274755000

6 -0.339203000 -14.996448000 24.531198000

6 -1.339639000 -15.714944000 23.874900000

6 1.004372000 -15.231405000 24.229622000

6 -0.989071000 -16.671079000 22.917973000

1 -2.385606000 -15.530989000 24.105452000

6 1.365597000 -16.192103000 23.277515000

1 1.782250000 -14.664215000 24.735785000

6 0.353489000 -16.909288000 22.623726000

1 -1.762888000 -17.234143000 22.402536000

6 2.825227000 -16.461531000 22.977461000

1 0.625938000 -17.651320000 21.877832000

7 3.082924000 -16.506771000 21.535344000

1 3.456096000 -15.702342000 23.462689000

1 3.130869000 -17.432958000 23.386998000

6 4.368776000 -16.831912000 21.110482000

1 2.679836000 -15.714809000 21.038081000

6 4.898432000 -18.229683000 21.134466000

6 4.646791000 -17.507950000 19.810269000

1 5.276720000 -15.951168000 21.452619000

1 5.931064000 -18.381661000 21.436412000

1 4.215851000 -19.030289000 21.411679000

1 3.796720000 -17.828069000 19.211882000

1 5.513032000 -17.183240000 19.240588000

^2^**TS**

26 6.466319000 -13.688608000 21.240012000

7 5.302173000 -13.007352000 22.746250000

6 5.668282000 -12.849713000 24.059887000

6 4.540467000 -12.389994000 24.833686000

6 3.491281000 -12.274663000 23.971763000

6 3.977004000 -12.669418000 22.671595000

1 4.568119000 -12.180078000 25.895525000

1 2.478265000 -11.953722000 24.178363000

6 3.196231000 -12.718042000 21.522459000

1 2.160170000 -12.404766000 21.607437000

7 4.915570000 -13.545858000 19.973438000

6 3.645242000 -13.115765000 20.270470000

6 2.824754000 -13.136583000 19.082993000

6 3.616657000 -13.572988000 18.065582000

6 4.923117000 -13.814926000 18.626754000

1 1.784157000 -12.839230000 19.049266000

1 3.361406000 -13.708898000 17.022274000

6 6.023124000 -14.229626000 17.889855000

1 5.876510000 -14.412476000 16.829957000

7 7.672224000 -14.183247000 19.700061000

6 7.305359000 -14.392356000 18.398841000

6 8.450821000 -14.798657000 17.618796000

6 9.511403000 -14.831928000 18.470000000

6 9.015144000 -14.450753000 19.771630000

1 8.426876000 -15.020528000 16.559428000

1 10.540848000 -15.090330000 18.256858000

6 9.796577000 -14.398181000 20.916536000

1 10.848947000 -14.643234000 20.812456000

7 8.055279000 -13.726870000 22.504405000

6 9.340561000 -14.069632000 22.184995000

6 10.168111000 -14.044445000 23.370062000

6 9.361421000 -13.685917000 24.404247000

6 8.040214000 -13.487465000 23.851788000

1 11.226014000 -14.273707000 23.385923000

1 9.617384000 -13.557158000 25.448281000

6 6.933932000 -13.079787000 24.583349000

1 7.071307000 -12.912648000 25.647185000

8 6.243961000 -15.307753000 21.671400000

16 7.102122000 -11.439012000 20.746148000

1 6.939047000 -10.929734000 21.987650000

1 -0.190288000 -14.510827000 26.086848000

6 -0.017095000 -15.188465000 25.254819000

6 -1.081972000 -15.894900000 24.693990000

6 1.273846000 -15.344610000 24.744269000

6 -0.849085000 -16.759418000 23.620947000

1 -2.087350000 -15.772094000 25.087934000

6 1.517785000 -16.214372000 23.675013000

1 2.099859000 -14.783664000 25.174709000

6 0.441626000 -16.919205000 23.117103000

1 -1.673888000 -17.312033000 23.178426000

6 2.925587000 -16.409802000 23.149905000

1 0.622960000 -17.589151000 22.280804000

7 2.976146000 -16.341820000 21.688148000

1 3.603513000 -15.668478000 23.599748000

1 3.308757000 -17.398097000 23.433580000

6 4.165660000 -16.706507000 21.053927000

1 2.568830000 -15.483174000 21.321674000

6 4.564418000 -18.140513000 20.876545000

6 4.189729000 -17.278750000 19.673839000

1 5.105620000 -15.988589000 21.306351000

1 5.611485000 -18.397720000 21.006546000

1 3.858147000 -18.904959000 21.191311000

1 3.234978000 -17.469239000 19.190504000

1 4.988863000 -16.964159000 19.008918000

^4^**PC**_reb_

26 -0.043898000 -0.335935000 0.281384000

7 1.924869000 -0.594594000 -0.044152000

6 2.871140000 0.390025000 -0.231314000

6 4.185802000 -0.191091000 -0.297054000

6 4.029881000 -1.539521000 -0.162787000

6 2.620487000 -1.783362000 -0.008138000

1 5.101045000 0.370769000 -0.434644000

1 4.789742000 -2.310273000 -0.172725000

6 2.057448000 -3.044946000 0.136047000

1 2.731002000 -3.896069000 0.141436000

7 -0.288250000 -2.342570000 0.302338000

6 0.700513000 -3.297070000 0.281847000

6 0.131657000 -4.611015000 0.458335000

6 -1.210901000 -4.440709000 0.601824000

6 -1.463663000 -3.023984000 0.508233000

1 0.701304000 -5.531488000 0.474664000

1 -1.973250000 -5.192968000 0.759745000

6 -2.719263000 -2.446180000 0.619114000

1 -3.563808000 -3.107099000 0.786482000

7 -2.037734000 -0.106140000 0.309596000

6 -2.978636000 -1.086208000 0.520518000

6 -4.295257000 -0.505986000 0.586906000

6 -4.144114000 0.835444000 0.403738000

6 -2.734933000 1.075761000 0.227199000

1 -5.207725000 -1.066261000 0.746507000

1 -4.906820000 1.603437000 0.382608000

6 -2.181535000 2.326011000 -0.011752000

1 -2.857423000 3.174652000 -0.045766000

7 0.163824000 1.624320000 -0.190631000

6 -0.830884000 2.573534000 -0.204519000

6 -0.268424000 3.883624000 -0.417908000

6 1.080054000 3.722700000 -0.513822000

6 1.342360000 2.313323000 -0.361676000

1 -0.844166000 4.798686000 -0.474948000

1 1.840567000 4.477885000 -0.666885000

6 2.607916000 1.745605000 -0.376210000

1 3.455513000 2.408882000 -0.517122000

8 -0.349183000 -0.444900000 -2.391625000

16 0.092876000 -0.057094000 2.700788000

1 1.407265000 -0.356372000 2.811504000

1 6.522291000 -2.886048000 -4.026106000

6 5.499675000 -3.126476000 -4.306084000

6 5.223473000 -4.300037000 -5.009073000

6 4.461345000 -2.261236000 -3.953263000

6 3.903491000 -4.601898000 -5.354583000

1 6.028901000 -4.976956000 -5.281924000

6 3.137125000 -2.550498000 -4.303250000

1 4.678915000 -1.354697000 -3.393194000

6 2.868989000 -3.733720000 -5.004977000

1 3.679680000 -5.516281000 -5.898482000

6 2.020553000 -1.581521000 -3.960187000

1 1.840260000 -3.969208000 -5.260785000

7 0.809395000 -2.276056000 -3.515263000

1 2.396610000 -0.850544000 -3.224180000

1 1.744473000 -1.009699000 -4.858094000

6 -0.355971000 -1.466920000 -3.383536000

1 0.989531000 -2.775933000 -2.645624000

6 -1.161820000 -1.189474000 -4.625977000

6 -1.687121000 -2.124633000 -3.544406000

1 0.261749000 0.260094000 -2.662752000

1 -1.632158000 -0.214076000 -4.709575000

1 -0.824215000 -1.628802000 -5.559876000

1 -1.694087000 -3.189588000 -3.753209000

1 -2.493996000 -1.760356000 -2.916564000

^2^**PC**_reb_

26 -0.050451000 -0.359490000 0.090613000

7 1.934783000 -0.641261000 -0.136608000

6 2.887194000 0.343763000 -0.252834000

6 4.207316000 -0.239879000 -0.279726000

6 4.044430000 -1.588637000 -0.181126000

6 2.625305000 -1.828555000 -0.082431000

1 5.128047000 0.323716000 -0.364511000

1 4.802176000 -2.361594000 -0.171491000

6 2.047237000 -3.083559000 0.071515000

1 2.710914000 -3.942449000 0.096058000

7 -0.287944000 -2.346570000 0.246991000

6 0.687388000 -3.316133000 0.241658000

6 0.103327000 -4.614831000 0.476402000

6 -1.232995000 -4.418056000 0.644165000

6 -1.468617000 -3.000844000 0.502173000

1 0.659315000 -5.543141000 0.512928000

1 -2.002336000 -5.153039000 0.844696000

6 -2.719577000 -2.404406000 0.592130000

1 -3.565990000 -3.052576000 0.798003000

7 -2.040764000 -0.087110000 0.154528000

6 -2.979358000 -1.050818000 0.421759000

6 -4.300144000 -0.465436000 0.462223000

6 -4.147691000 0.862882000 0.212099000

6 -2.733309000 1.090531000 0.027321000

1 -5.212534000 -1.016061000 0.653618000

1 -4.907864000 1.631904000 0.158333000

6 -2.165132000 2.333161000 -0.218028000

1 -2.833176000 3.186257000 -0.286607000

7 0.179503000 1.613733000 -0.277740000

6 -0.803173000 2.573219000 -0.340410000

6 -0.216282000 3.879955000 -0.509532000

6 1.133909000 3.705088000 -0.526810000

6 1.373251000 2.289874000 -0.376939000

1 -0.778101000 4.801739000 -0.592689000

1 1.909431000 4.453577000 -0.630190000

6 2.632998000 1.705138000 -0.361344000

1 3.489830000 2.366133000 -0.450456000

8 -0.298063000 -0.426595000 -2.162190000

16 0.121400000 -0.064707000 2.300528000

1 0.961206000 -1.091261000 2.567378000

1 6.529406000 -2.763114000 -4.153774000

6 5.498848000 -3.012963000 -4.393225000

6 5.207165000 -4.180030000 -5.100313000

6 4.466080000 -2.165852000 -3.983688000

6 3.877233000 -4.494772000 -5.393252000

1 6.008411000 -4.842493000 -5.417362000

6 3.132129000 -2.468028000 -4.280362000

1 4.696113000 -1.265371000 -3.419177000

6 2.848482000 -3.645168000 -4.986850000

1 3.641666000 -5.404449000 -5.940019000

6 2.017088000 -1.523008000 -3.872630000

1 1.812935000 -3.892491000 -5.201448000

7 0.867748000 -2.245489000 -3.318763000

1 2.424208000 -0.765957000 -3.181938000

1 1.653539000 -0.981259000 -4.757692000

6 -0.317094000 -1.491784000 -3.127494000

1 1.127410000 -2.729288000 -2.460931000

6 -1.215017000 -1.255668000 -4.313559000

6 -1.632707000 -2.195074000 -3.191855000

1 0.274538000 0.294612000 -2.473304000

1 -1.723976000 -0.298369000 -4.374790000

1 -0.918505000 -1.691122000 -5.262970000

1 -1.608932000 -3.262247000 -3.386758000

1 -2.409741000 -1.856020000 -2.515131000

^4^**PC**_DHA_

26 -0.045289000 -0.632723000 0.420365000

7 1.494474000 -1.944199000 0.363029000

6 2.826108000 -1.644963000 0.212959000

6 3.624992000 -2.834947000 0.376614000

6 2.761468000 -3.854398000 0.642887000

6 1.433791000 -3.288268000 0.635188000

1 4.705039000 -2.865944000 0.306939000

1 2.986928000 -4.894444000 0.843849000

6 0.273679000 -4.010458000 0.878870000

1 0.378014000 -5.070352000 1.089812000

7 -1.335732000 -2.165558000 0.648200000

6 -1.010546000 -3.481661000 0.882421000

6 -2.204131000 -4.251549000 1.118708000

6 -3.256218000 -3.389630000 1.024486000

6 -2.707106000 -2.090723000 0.733523000

1 -2.220591000 -5.313354000 1.329993000

1 -4.311846000 -3.599794000 1.141670000

6 -3.466399000 -0.940965000 0.553348000

1 -4.543850000 -1.032824000 0.648067000

7 -1.618605000 0.614643000 0.124333000

6 -2.951007000 0.316621000 0.270546000

6 -3.749456000 1.506478000 0.097928000

6 -2.883500000 2.528485000 -0.143331000

6 -1.555394000 1.964076000 -0.119732000

1 -4.829783000 1.538086000 0.162048000

1 -3.105523000 3.573480000 -0.318947000

6 -0.391402000 2.695168000 -0.306251000

1 -0.493242000 3.759220000 -0.495753000

7 1.222237000 0.857782000 -0.044407000

6 0.895078000 2.171655000 -0.278264000

6 2.087585000 2.942527000 -0.523253000

6 3.138973000 2.077645000 -0.448409000

6 2.589426000 0.777996000 -0.156862000

1 2.102216000 4.005391000 -0.729180000

1 4.193466000 2.284948000 -0.580072000

6 3.344379000 -0.381313000 -0.032757000

1 4.421079000 -0.291222000 -0.136794000

8 -0.313102000 -0.901504000 -2.037844000

16 0.055639000 -0.279195000 2.845298000

1 1.078391000 0.604905000 2.815743000

1 2.906240000 1.792545000 -3.889031000

6 2.946941000 0.917689000 -4.532672000

6 4.008500000 0.749751000 -5.426523000

6 1.932922000 -0.037056000 -4.455677000

6 4.054406000 -0.382898000 -6.239858000

1 4.794858000 1.497704000 -5.487949000

6 1.974692000 -1.179988000 -5.267872000

1 1.113489000 0.095846000 -3.754326000

6 3.043910000 -1.344051000 -6.155265000

1 4.875513000 -0.522052000 -6.938454000

6 0.878201000 -2.227466000 -5.190061000

1 3.085668000 -2.227493000 -6.788957000

7 0.795253000 -2.821083000 -3.842014000

1 -0.095875000 -1.761866000 -5.386985000

1 1.042196000 -2.999359000 -5.954218000

6 1.155753000 -4.021675000 -3.701053000

1 0.111686000 -1.660885000 -2.510177000

6 1.687767000 -5.271599000 -4.272836000

6 1.353512000 -5.119181000 -2.759642000

1 -1.267134000 -1.066340000 -2.087697000

1 1.032731000 -5.900175000 -4.874076000

1 2.736420000 -5.313455000 -4.562515000

1 2.179247000 -5.049006000 -2.053985000

1 0.481352000 -5.647231000 -2.377902000

^2^**PC**_DHA_

26 -0.064464000 -0.667317000 0.256891000

7 1.474715000 -1.967302000 0.303146000

6 2.810418000 -1.696097000 0.130355000

6 3.593804000 -2.888247000 0.347704000

6 2.716510000 -3.880511000 0.667284000

6 1.395403000 -3.296890000 0.634260000

1 4.673022000 -2.938606000 0.274739000

1 2.927813000 -4.913685000 0.914716000

6 0.223825000 -4.001817000 0.886105000

1 0.322511000 -5.053523000 1.138879000

7 -1.384437000 -2.171640000 0.565089000

6 -1.061239000 -3.475865000 0.850348000

6 -2.258788000 -4.239418000 1.111169000

6 -3.308437000 -3.381040000 0.989687000

6 -2.753561000 -2.090290000 0.659943000

1 -2.275641000 -5.293277000 1.359861000

1 -4.364358000 -3.583687000 1.117799000

6 -3.500155000 -0.931079000 0.488045000

1 -4.578303000 -1.007549000 0.590400000

7 -1.629352000 0.597671000 0.070722000

6 -2.965647000 0.324301000 0.224015000

6 -3.743234000 1.532614000 0.077666000

6 -2.859052000 2.539843000 -0.156646000

6 -1.541054000 1.947386000 -0.158177000

1 -4.822228000 1.584325000 0.151564000

1 -3.061624000 3.591422000 -0.316503000

6 -0.367573000 2.656307000 -0.377232000

1 -0.458815000 3.724566000 -0.549656000

7 1.232404000 0.799524000 -0.201346000

6 0.913186000 2.117736000 -0.400564000

6 2.110117000 2.885798000 -0.658226000

6 3.154664000 2.013686000 -0.612176000

6 2.595999000 0.713343000 -0.324117000

1 2.129098000 3.952332000 -0.844586000

1 4.209435000 2.214389000 -0.752130000

6 3.340962000 -0.449596000 -0.176754000

1 4.418025000 -0.375176000 -0.290870000

8 -0.318066000 -0.969320000 -1.839622000

16 0.074199000 -0.242243000 2.456749000

1 1.421590000 -0.172927000 2.554521000

1 2.929190000 1.904016000 -3.840737000

6 2.941200000 1.018945000 -4.470689000

6 3.962377000 0.832384000 -5.406658000

6 1.930521000 0.067365000 -4.333910000

6 3.971274000 -0.313976000 -6.201962000

1 4.746308000 1.577431000 -5.515250000

6 1.934430000 -1.088804000 -5.127421000

1 1.140678000 0.217393000 -3.602865000

6 2.963623000 -1.270739000 -6.057870000

1 4.760726000 -0.466385000 -6.933438000

6 0.842662000 -2.132688000 -4.981951000

1 2.974723000 -2.164246000 -6.678536000

7 0.865510000 -2.755336000 -3.643134000

1 -0.141619000 -1.660155000 -5.089671000

1 0.941004000 -2.891107000 -5.769885000

6 1.238585000 -3.957826000 -3.556066000

1 0.214359000 -1.677901000 -2.298290000

6 1.727737000 -5.190114000 -4.199113000

6 1.504688000 -5.079206000 -2.662243000

1 -1.243867000 -1.254444000 -1.906548000

1 1.030788000 -5.802477000 -4.769109000

1 2.752419000 -5.220451000 -4.565663000

1 2.378085000 -5.023910000 -2.015445000

1 0.661409000 -5.616120000 -2.231368000

- 1. **N-HAT→Ring-opening/Orebound.**

^4^**RC**

26 6.414209000 -13.780820000 21.458996000

7 5.037305000 -13.564796000 22.916413000

6 5.247794000 -13.080286000 24.180374000

6 4.038227000 -13.171392000 24.962371000

6 3.093382000 -13.721348000 24.152947000

6 3.730852000 -13.962610000 22.881115000

1 3.944017000 -12.851999000 25.992224000

1 2.059731000 -13.950200000 24.377531000

6 3.089231000 -14.513227000 21.775693000

1 2.046440000 -14.790526000 21.893069000

7 4.979165000 -14.464949000 20.215781000

6 3.673521000 -14.746129000 20.540181000

6 2.991822000 -15.331050000 19.411092000

6 3.901324000 -15.407167000 18.401996000

6 5.136371000 -14.864715000 18.914020000

1 1.954139000 -15.638525000 19.410237000

1 3.765534000 -15.787677000 17.397856000

6 6.311691000 -14.761182000 18.179890000

1 6.293167000 -15.123670000 17.156722000

7 7.706186000 -13.722612000 19.909152000

6 7.498520000 -14.215013000 18.639879000

6 8.702820000 -14.097203000 17.855424000

6 9.647004000 -13.543139000 18.662285000

6 9.024059000 -13.322963000 19.944331000

1 8.795235000 -14.406009000 16.822131000

1 10.676728000 -13.303193000 18.430214000

6 9.669778000 -12.801420000 21.053584000

1 10.717646000 -12.539683000 20.942194000

7 7.779856000 -12.843518000 22.612299000

6 9.085706000 -12.578815000 22.296799000

6 9.773550000 -12.011950000 23.430976000

6 8.863988000 -11.938822000 24.441130000

6 7.625476000 -12.464022000 23.920927000

1 10.813850000 -11.712894000 23.435257000

1 9.001446000 -11.567670000 25.448604000

6 6.448284000 -12.565085000 24.651554000

1 6.470681000 -12.217614000 25.680013000

8 6.859999000 -15.267777000 21.962838000

16 5.775779000 -11.544574000 20.419646000

1 6.949462000 -10.903399000 20.620287000

1 2.166958000 -22.376337000 21.746989000

6 2.203885000 -21.346416000 22.093640000

6 1.110644000 -20.793112000 22.762849000

6 3.352445000 -20.581991000 21.878009000

6 1.177919000 -19.472312000 23.213467000

1 0.217395000 -21.387475000 22.937738000

6 3.426495000 -19.254113000 22.315951000

1 4.205368000 -21.022318000 21.364960000

6 2.325427000 -18.709865000 22.990576000

1 0.333816000 -19.035992000 23.742764000

6 4.658737000 -18.411418000 22.020758000

1 2.393487000 -17.686485000 23.347895000

7 4.943742000 -17.423293000 23.057993000

1 5.512245000 -19.088256000 21.831895000

1 4.489104000 -17.856250000 21.087819000

6 5.531313000 -17.971599000 24.255370000

1 5.540544000 -16.687672000 22.679408000

6 6.980569000 -18.426686000 24.311757000

6 6.535210000 -17.162250000 25.021039000

1 4.825490000 -18.557988000 24.841575000

1 7.236849000 -19.298797000 24.909073000

1 7.580146000 -18.301077000 23.413302000

1 6.846903000 -16.215821000 24.585437000

1 6.481880000 -17.161441000 26.107019000

^2^**RC**

26 6.429512000 -13.781451000 21.455941000

7 5.055765000 -13.455052000 22.894363000

6 5.268874000 -12.915008000 24.135342000

6 4.059653000 -12.963862000 24.920465000

6 3.111966000 -13.550771000 24.140087000

6 3.744726000 -13.856367000 22.880879000

1 3.967507000 -12.595364000 25.934027000

1 2.078446000 -13.766057000 24.378399000

6 3.105022000 -14.465106000 21.809648000

1 2.062116000 -14.735742000 21.940717000

7 4.990326000 -14.493797000 20.244982000

6 3.688415000 -14.754238000 20.582027000

6 2.992139000 -15.358223000 19.472280000

6 3.891843000 -15.454010000 18.454908000

6 5.134359000 -14.914278000 18.950977000

1 1.952834000 -15.659749000 19.486582000

1 3.745264000 -15.850102000 17.458322000

6 6.308362000 -14.840182000 18.208008000

1 6.274763000 -15.211115000 17.188115000

7 7.743767000 -13.840249000 19.922545000

6 7.521314000 -14.349171000 18.666546000

6 8.737763000 -14.320433000 17.891236000

6 9.702674000 -13.802404000 18.697980000

6 9.072065000 -13.504724000 19.961448000

1 8.823580000 -14.659601000 16.866923000

1 10.746556000 -13.624055000 18.474114000

6 9.725032000 -12.937962000 21.049065000

1 10.784855000 -12.729280000 20.940181000

7 7.803049000 -12.789298000 22.564574000

6 9.128384000 -12.589619000 22.249808000

6 9.815073000 -11.966572000 23.354650000

6 8.899166000 -11.801194000 24.346337000

6 7.650691000 -12.329410000 23.853606000

1 10.863485000 -11.697383000 23.353042000

1 9.039399000 -11.370519000 25.329484000

6 6.475093000 -12.386724000 24.584931000

1 6.498502000 -11.993111000 25.596580000

8 6.858413000 -15.244736000 22.032968000

16 6.108339000 -11.388888000 20.601029000

1 5.678913000 -10.837585000 21.758963000

1 2.269009000 -22.406847000 21.626069000

6 2.277513000 -21.378793000 21.980057000

6 1.158336000 -20.851575000 22.627011000

6 3.415491000 -20.590551000 21.796202000

6 1.189085000 -19.532741000 23.087389000

1 0.273141000 -21.464441000 22.777249000

6 3.453315000 -19.264571000 22.244336000

1 4.288686000 -21.010589000 21.300557000

6 2.326230000 -18.746568000 22.896317000

1 0.324485000 -19.116588000 23.599570000

6 4.675417000 -18.395897000 21.984864000

1 2.365819000 -17.724644000 23.262023000

7 4.921116000 -17.416884000 23.040968000

1 5.545022000 -19.054453000 21.805129000

1 4.514145000 -17.831441000 21.056111000

6 5.491628000 -17.973755000 24.242932000

1 5.516078000 -16.668355000 22.684972000

6 6.945027000 -18.411448000 24.322562000

6 6.469234000 -17.163014000 25.040462000

1 4.781866000 -18.577476000 24.806365000

1 7.200151000 -19.289008000 24.912355000

1 7.561275000 -18.264843000 23.438680000

1 6.778591000 -16.207091000 24.624488000

1 6.393705000 -17.178312000 26.125008000

^4^**TS**

26 6.396541000 -13.893223000 21.520611000

7 5.053883000 -13.621856000 23.013715000

6 5.306614000 -13.129267000 24.267457000

6 4.092898000 -13.126300000 25.051052000

6 3.107681000 -13.618808000 24.250221000

6 3.719163000 -13.928346000 22.978637000

1 4.020594000 -12.778271000 26.073912000

1 2.059025000 -13.760375000 24.480243000

6 3.041456000 -14.476349000 21.895714000

1 1.975797000 -14.651111000 22.012216000

7 4.926596000 -14.635681000 20.331498000

6 3.610000000 -14.798915000 20.667466000

6 2.885978000 -15.380954000 19.560577000

6 3.787307000 -15.562452000 18.554706000

6 5.059789000 -15.085454000 19.044445000

1 1.826876000 -15.606364000 19.566030000

1 3.619794000 -15.964402000 17.563290000

6 6.232649000 -15.067038000 18.299832000

1 6.188066000 -15.455255000 17.286868000

7 7.690657000 -14.025224000 19.973004000

6 7.450201000 -14.563417000 18.739398000

6 8.651482000 -14.516649000 17.935080000

6 9.613821000 -13.940896000 18.703734000

6 9.002215000 -13.640348000 19.979575000

1 8.724796000 -14.876519000 16.916518000

1 10.645383000 -13.730489000 18.451113000

6 9.671169000 -13.061424000 21.049320000

1 10.717380000 -12.809295000 20.905916000

7 7.817322000 -13.027343000 22.656550000

6 9.114109000 -12.786490000 22.290813000

6 9.841628000 -12.210009000 23.398888000

6 8.966716000 -12.114475000 24.435956000

6 7.701440000 -12.627354000 23.961317000

1 10.885050000 -11.922838000 23.366524000

1 9.140073000 -11.730349000 25.433385000

6 6.540322000 -12.677280000 24.721633000

1 6.594824000 -12.312765000 25.742947000

8 6.809658000 -15.441131000 22.070196000

16 5.807061000 -11.736103000 20.676941000

1 5.785591000 -11.087550000 21.863084000

1 2.590847000 -22.309361000 22.275716000

6 2.546561000 -21.224558000 22.319912000

6 1.386946000 -20.586939000 22.761514000

6 3.655833000 -20.467850000 21.933182000

6 1.339269000 -19.190611000 22.812889000

1 0.523183000 -21.173214000 23.062946000

6 3.614740000 -19.069962000 21.978106000

1 4.557673000 -20.968168000 21.587169000

6 2.444809000 -18.436414000 22.422586000

1 0.437206000 -18.689538000 23.153808000

6 4.807787000 -18.240460000 21.540279000

1 2.409144000 -17.350602000 22.459409000

7 5.372504000 -17.410922000 22.595289000

1 5.600635000 -18.903097000 21.156383000

1 4.537641000 -17.562380000 20.724523000

6 5.654340000 -17.927716000 23.851695000

1 5.919588000 -16.445229000 22.304919000

6 7.023282000 -17.557663000 24.493076000

6 5.789292000 -16.944068000 25.031649000

1 5.253669000 -18.913682000 24.064859000

1 7.504022000 -18.367216000 25.035809000

1 7.664223000 -16.938727000 23.872355000

1 5.598260000 -15.901437000 24.799752000

1 5.378550000 -17.300892000 25.972294000

^2^**TS**

26 6.383541000 -13.909743000 21.483488000

7 5.013333000 -13.668474000 22.959942000

6 5.243625000 -13.198516000 24.226802000

6 4.016860000 -13.214635000 24.989829000

6 3.047015000 -13.694535000 24.163152000

6 3.681755000 -13.978651000 22.896880000

1 3.926013000 -12.885774000 26.017535000

1 1.994921000 -13.842926000 24.372196000

6 3.025287000 -14.508242000 21.791124000

1 1.958415000 -14.689028000 21.883985000

7 4.942591000 -14.640044000 20.262085000

6 3.618659000 -14.810158000 20.570569000

6 2.918699000 -15.380783000 19.442856000

6 3.839968000 -15.549172000 18.453229000

6 5.101118000 -15.074809000 18.972532000

1 1.860509000 -15.609782000 19.425679000

1 3.693315000 -15.941345000 17.454662000

6 6.287320000 -15.043788000 18.249929000

1 6.263459000 -15.421877000 17.232504000

7 7.705003000 -14.008441000 19.960570000

6 7.492325000 -14.535575000 18.716622000

6 8.707008000 -14.467903000 17.934919000

6 9.649178000 -13.889820000 18.726538000

6 9.012712000 -13.609534000 19.994512000

1 8.802279000 -14.816169000 16.914163000

1 10.682740000 -13.665924000 18.494519000

6 9.658459000 -13.037077000 21.081548000

1 10.704432000 -12.773713000 20.958081000

7 7.779068000 -13.042284000 22.657710000

6 9.079313000 -12.785187000 22.317900000

6 9.784118000 -12.223592000 23.448063000

6 8.892004000 -12.156020000 24.472768000

6 7.638993000 -12.670248000 23.967895000

1 10.825504000 -11.927532000 23.438189000

1 9.046222000 -11.790351000 25.480222000

6 6.466307000 -12.746003000 24.708906000

1 6.501764000 -12.400781000 25.737744000

8 6.809715000 -15.452360000 22.042903000

16 5.795414000 -11.732179000 20.673912000

1 5.787224000 -11.098617000 21.868083000

1 2.436453000 -22.226990000 22.260118000

6 2.437708000 -21.145301000 22.363633000

6 1.335199000 -20.491801000 22.914637000

6 3.549015000 -20.409049000 21.944611000

6 1.346959000 -19.099692000 23.042995000

1 0.470316000 -21.062231000 23.242161000

6 3.566386000 -19.015176000 22.064813000

1 4.406234000 -20.922095000 21.514172000

6 2.453872000 -18.365458000 22.619778000

1 0.490071000 -18.585793000 23.470481000

6 4.759443000 -18.206881000 21.586161000

1 2.463897000 -17.282927000 22.717630000

7 5.375527000 -17.395886000 22.626308000

1 5.522047000 -18.884832000 21.169315000

1 4.471618000 -17.519167000 20.784720000

6 5.748655000 -17.959104000 23.839925000

1 5.923534000 -16.423979000 22.320137000

6 7.158650000 -17.607453000 24.395926000

6 5.962748000 -17.022371000 25.043682000

1 5.369495000 -18.956251000 24.041873000

1 7.678771000 -18.434231000 24.872339000

1 7.752924000 -16.962360000 23.755903000

1 5.752730000 -15.972276000 24.866538000

1 5.623173000 -17.416179000 25.997897000

^4^**IM**

26 7.507182000 -14.501462000 21.785434000

7 5.521512000 -14.442485000 22.178733000

6 4.933183000 -14.252305000 23.402578000

6 3.497786000 -14.229162000 23.260481000

6 3.230496000 -14.385964000 21.933886000

6 4.501912000 -14.511377000 21.263621000

1 2.801780000 -14.090198000 24.078104000

1 2.269428000 -14.404505000 21.435696000

6 4.652675000 -14.659019000 19.892184000

1 3.750901000 -14.702504000 19.289718000

7 7.108119000 -14.700006000 19.820038000

6 5.870101000 -14.753030000 19.231715000

6 6.010412000 -14.961436000 17.811572000

6 7.344743000 -15.049010000 17.555669000

6 8.024509000 -14.889421000 18.817490000

1 5.183055000 -15.036116000 17.117350000

1 7.841437000 -15.209347000 16.607217000

6 9.402559000 -14.936569000 18.974215000

1 10.002276000 -15.093088000 18.083329000

7 9.485027000 -14.573865000 21.398361000

6 10.071412000 -14.783859000 20.179120000

6 11.509111000 -14.787161000 20.317480000

6 11.777667000 -14.562240000 21.631734000

6 10.504172000 -14.425570000 22.299748000

1 12.204255000 -14.936026000 19.501013000

1 12.739796000 -14.488778000 22.122635000

6 10.354751000 -14.167927000 23.654270000

1 11.256711000 -14.076068000 24.250889000

7 7.898693000 -14.112356000 23.725052000

6 9.138150000 -14.034344000 24.308306000

6 9.000306000 -13.827238000 25.729001000

6 7.665845000 -13.805141000 25.997835000

6 6.983399000 -13.994665000 24.741411000

1 9.829646000 -13.725539000 26.417395000

1 7.171017000 -13.679592000 26.952572000

6 5.603563000 -14.058454000 24.602394000

1 5.003269000 -13.927726000 25.497217000

8 7.547644000 -16.247599000 22.052347000

16 7.474832000 -12.216635000 21.328405000

1 7.613544000 -11.768744000 22.596112000

1 6.656134000 -16.683515000 22.134704000

1 7.493357000 -19.907298000 24.305577000

6 7.094133000 -18.937763000 24.019601000

6 5.984565000 -18.349755000 24.811322000

1 7.790562000 -18.261838000 23.533477000

6 5.663918000 -18.952650000 23.433963000

1 5.920808000 -17.268073000 24.875401000

1 5.594267000 -18.892422000 25.668161000

7 5.407299000 -18.060331000 22.394108000

1 5.150044000 -19.914505000 23.440756000

6 4.549277000 -18.562070000 21.328422000

6 3.188134000 -19.071472000 21.783536000

1 5.078237000 -19.374510000 20.801662000

1 4.422745000 -17.746786000 20.607059000

6 2.779215000 -20.382810000 21.515178000

6 2.313542000 -18.218996000 22.474451000

6 1.520986000 -20.836460000 21.920428000

1 3.447682000 -21.053488000 20.979621000

6 1.059441000 -18.671127000 22.883186000

1 2.622469000 -17.199123000 22.689520000

1 1.218301000 -21.857069000 21.700889000

6 0.658342000 -19.981517000 22.606559000

1 0.391377000 -17.999684000 23.416725000

1 -0.320258000 -20.331906000 22.923872000

^2^**IM**

26 7.476970000 -14.473244000 21.821401000

7 5.473788000 -14.391213000 22.114273000

6 4.828158000 -14.177037000 23.304707000

6 3.401578000 -14.147465000 23.092027000

6 3.198346000 -14.323356000 21.756219000

6 4.499957000 -14.468875000 21.151429000

1 2.667224000 -13.989657000 23.871855000

1 2.262401000 -14.345877000 21.212484000

6 4.717748000 -14.650537000 19.792757000

1 3.847101000 -14.694981000 19.146127000

7 7.172950000 -14.740977000 19.848095000

6 5.964860000 -14.786827000 19.198764000

6 6.172503000 -15.044489000 17.795184000

6 7.515740000 -15.169752000 17.611246000

6 8.134399000 -14.980293000 18.899885000

1 5.379962000 -15.124603000 17.062048000

1 8.055902000 -15.371212000 16.694950000

6 9.502649000 -15.035333000 19.126340000

1 10.143630000 -15.233519000 18.273292000

7 9.469596000 -14.562374000 21.532640000

6 10.113373000 -14.830309000 20.354086000

6 11.542833000 -14.825854000 20.561171000

6 11.748625000 -14.536291000 21.874040000

6 10.445006000 -14.370547000 22.473767000

1 12.276060000 -15.012929000 19.786960000

1 12.686366000 -14.438011000 22.406028000

6 10.231673000 -14.059352000 23.808109000

1 11.104329000 -13.939165000 24.442102000

7 7.775250000 -14.023088000 23.763647000

6 8.984931000 -13.915092000 24.400663000

6 8.779495000 -13.670420000 25.807312000

6 7.433888000 -13.658048000 26.013962000

6 6.812188000 -13.887944000 24.732975000

1 9.575008000 -13.540406000 26.530002000

1 6.894071000 -13.512764000 26.941128000

6 5.441197000 -13.960883000 24.530968000

1 4.799242000 -13.815009000 25.393912000

8 7.488306000 -16.208047000 22.156197000

16 7.471195000 -12.204374000 21.293293000

1 7.425869000 -11.717919000 22.553510000

1 6.604778000 -16.662128000 22.100644000

1 7.716372000 -20.294977000 23.365401000

6 7.338920000 -19.278365000 23.292977000

6 6.458954000 -18.750701000 24.365416000

1 7.968923000 -18.570530000 22.764356000

6 5.819438000 -19.151511000 23.027893000

1 6.496194000 -17.684723000 24.565929000

1 6.214380000 -19.380427000 25.216643000

7 5.411539000 -18.123949000 22.178007000

1 5.258420000 -20.086721000 23.027532000

6 4.348130000 -18.470153000 21.241193000

6 3.066001000 -18.969454000 21.893398000

1 4.722032000 -19.243023000 20.548564000

1 4.142237000 -17.576007000 20.643167000

6 2.606724000 -20.275271000 21.684947000

6 2.322834000 -18.116814000 22.723596000

6 1.428599000 -20.724245000 22.287992000

1 3.171257000 -20.944877000 21.039535000

6 1.148686000 -18.563806000 23.328496000

1 2.668092000 -17.099486000 22.889644000

1 1.084825000 -21.740252000 22.111933000

6 0.697355000 -19.869251000 23.112715000

1 0.581659000 -17.891850000 23.967832000

1 -0.219265000 -20.215107000 23.583139000

^4^**TS**_ring_

26 7.504665000 -14.483462000 21.769006000

7 5.507862000 -14.371859000 22.085248000

6 4.877513000 -14.172127000 23.286623000

6 3.449242000 -14.118762000 23.090247000

6 3.229292000 -14.266131000 21.754102000

6 4.522778000 -14.415391000 21.131998000

1 2.725809000 -13.966155000 23.881218000

1 2.287736000 -14.261893000 21.219550000

6 4.722942000 -14.558934000 19.766315000

1 3.844424000 -14.578112000 19.129186000

7 7.177102000 -14.663328000 19.789433000

6 5.962213000 -14.680397000 19.153008000

6 6.152602000 -14.884376000 17.737935000

6 7.493093000 -15.005660000 17.533711000

6 8.126946000 -14.870980000 18.822216000

1 5.351357000 -14.933549000 17.011559000

1 8.022130000 -15.173711000 16.604266000

6 9.496029000 -14.955589000 19.032044000

1 10.125671000 -15.123374000 18.164121000

7 9.494184000 -14.606821000 21.459413000

6 10.121694000 -14.826929000 20.263022000

6 11.552383000 -14.870515000 20.457454000

6 11.775133000 -14.659641000 21.782590000

6 10.480527000 -14.490880000 22.401057000

1 12.274851000 -15.033996000 19.667922000

1 12.718964000 -14.614530000 22.311037000

6 10.285110000 -14.233092000 23.749745000

1 11.165299000 -14.167064000 24.381362000

7 7.830543000 -14.110092000 23.725288000

6 9.047984000 -14.066143000 24.356615000

6 8.860138000 -13.853836000 25.770713000

6 7.516948000 -13.793164000 25.986753000

6 6.879224000 -13.964426000 24.704579000

1 9.664165000 -13.774458000 26.491371000

1 6.988784000 -13.651654000 26.921220000

6 5.504942000 -13.992858000 24.510996000

1 4.873384000 -13.849615000 25.382027000

8 7.490888000 -16.228948000 22.029617000

16 7.558557000 -12.195365000 21.320644000

1 7.712683000 -11.753823000 22.588870000

1 6.581665000 -16.640245000 22.105675000

1 7.259125000 -20.342436000 23.909935000

6 7.257862000 -19.284019000 23.674874000

6 6.531997000 -18.305074000 24.511659000

1 7.859964000 -18.926060000 22.849097000

6 5.477562000 -18.641644000 23.498183000

1 6.895762000 -17.281087000 24.446434000

1 6.297322000 -18.614135000 25.531140000

7 5.275804000 -17.877387000 22.440925000

1 4.834777000 -19.495930000 23.721520000

6 4.300945000 -18.304557000 21.432702000

6 3.054684000 -19.007100000 21.941396000

1 4.822336000 -18.954497000 20.712148000

1 4.016550000 -17.402969000 20.879101000

6 2.847724000 -20.374618000 21.724978000

6 2.083121000 -18.284348000 22.651161000

6 1.696602000 -21.009361000 22.200503000

1 3.590927000 -20.946433000 21.173132000

6 0.934414000 -18.914190000 23.128133000

1 2.234960000 -17.221161000 22.824124000

1 1.550764000 -22.071198000 22.019506000

6 0.737399000 -20.280188000 22.903524000

1 0.188631000 -18.339775000 23.671844000

1 -0.159520000 -20.770489000 23.272679000

^2^**TS**_ring_

26 7.505634000 -14.478067000 21.780310000

7 5.507752000 -14.365711000 22.094631000

6 4.876574000 -14.160022000 23.294388000

6 3.448382000 -14.108347000 23.096725000

6 3.229521000 -14.262270000 21.761100000

6 4.523547000 -14.414718000 21.140850000

1 2.724261000 -13.952066000 23.886348000

1 2.288345000 -14.261261000 21.225865000

6 4.725077000 -14.568309000 19.776391000

1 3.847219000 -14.590873000 19.138450000

7 7.179218000 -14.678879000 19.802753000

6 5.964641000 -14.698346000 19.165464000

6 6.155645000 -14.916911000 17.752697000

6 7.495927000 -15.044821000 17.550880000

6 8.129152000 -14.898729000 18.838385000

1 5.354873000 -14.970758000 17.026131000

1 8.025166000 -15.223729000 16.623579000

6 9.498124000 -14.981945000 19.050052000

1 10.128272000 -15.160298000 18.184610000

7 9.494656000 -14.601415000 21.472732000

6 10.123014000 -14.836675000 20.279496000

6 11.553705000 -14.874343000 20.474828000

6 11.775736000 -14.644089000 21.796849000

6 10.480686000 -14.470180000 22.412857000

1 12.276709000 -15.047103000 19.687765000

1 12.719375000 -14.589315000 22.324720000

6 10.284519000 -14.196907000 23.758333000

1 11.164515000 -14.120318000 24.389018000

7 7.829630000 -14.085479000 23.732950000

6 9.046695000 -14.029624000 24.363693000

6 8.857893000 -13.806534000 25.776068000

6 7.514449000 -13.752147000 25.991855000

6 6.877539000 -13.937270000 24.711173000

1 9.661572000 -13.716671000 26.495880000

1 6.985575000 -13.606170000 26.925232000

6 5.503447000 -13.972205000 24.517872000

1 4.871421000 -13.825304000 25.387955000

8 7.491336000 -16.220784000 22.060965000

16 7.551898000 -12.194564000 21.310959000

1 7.668995000 -11.740426000 22.578666000

1 6.583332000 -16.636961000 22.115189000

1 7.263571000 -20.390330000 23.838942000

6 7.267192000 -19.328766000 23.618509000

6 6.557041000 -18.356379000 24.475960000

1 7.864033000 -18.963865000 22.791989000

6 5.490412000 -18.672215000 23.468458000

1 6.927524000 -17.334177000 24.419616000

1 6.330545000 -18.677624000 25.493523000

7 5.282347000 -17.891593000 22.424298000

1 4.844514000 -19.525551000 23.686438000

6 4.294079000 -18.299657000 21.421016000

6 3.047825000 -18.999019000 21.934082000

1 4.803175000 -18.945083000 20.687704000

1 4.010848000 -17.389641000 20.880954000

6 2.830516000 -20.363259000 21.707212000

6 2.086897000 -18.276810000 22.658708000

6 1.679768000 -20.995304000 22.187182000

1 3.565204000 -20.934575000 21.143556000

6 0.938493000 -18.903956000 23.139999000

1 2.246618000 -17.216114000 22.839706000

1 1.525847000 -22.054568000 21.997962000

6 0.731187000 -20.266680000 22.905028000

1 0.200958000 -18.329917000 23.695234000

1 -0.165486000 -20.754836000 23.277608000

^4^**PC**_ring_

26 7.530857000 -14.484621000 21.688955000

7 5.535941000 -14.406727000 22.033076000

6 4.916385000 -14.279732000 23.250316000

6 3.487059000 -14.209726000 23.070021000

6 3.255136000 -14.270947000 21.728665000

6 4.542429000 -14.384525000 21.086880000

1 2.770896000 -14.103284000 23.875096000

1 2.309179000 -14.226038000 21.203687000

6 4.730017000 -14.437878000 19.712925000

1 3.845297000 -14.418481000 19.084446000

7 7.184671000 -14.525681000 19.704783000

6 5.963316000 -14.510669000 19.080663000

6 6.140619000 -14.615454000 17.653125000

6 7.479916000 -14.708462000 17.427894000

6 8.125964000 -14.656510000 18.716364000

1 5.332395000 -14.622648000 16.932884000

1 8.000869000 -14.807351000 16.484075000

6 9.497751000 -14.743752000 18.906165000

1 10.119668000 -14.844870000 18.022523000

7 9.517304000 -14.572093000 21.351950000

6 10.134507000 -14.700803000 20.137218000

6 11.567039000 -14.754861000 20.315500000

6 11.800916000 -14.644633000 21.650725000

6 10.511439000 -14.525824000 22.291245000

1 12.282799000 -14.857358000 19.509793000

1 12.749147000 -14.638179000 22.173095000

6 10.327931000 -14.368729000 23.656920000

1 11.213766000 -14.348381000 24.283698000

7 7.873138000 -14.246196000 23.663883000

6 9.096589000 -14.245575000 24.285458000

6 8.922171000 -14.127808000 25.712269000

6 7.581361000 -14.078708000 25.944249000

6 6.931444000 -14.164415000 24.659611000

1 9.732940000 -14.097874000 26.429069000

1 7.062245000 -13.997929000 26.890935000

6 5.555511000 -14.178887000 24.477960000

1 4.932350000 -14.088575000 25.362101000

8 7.543513000 -16.245514000 21.823036000

16 7.563183000 -12.170597000 21.403973000

1 7.739199000 -11.818617000 22.697116000

1 6.654876000 -16.680403000 21.941164000

1 6.938659000 -20.140177000 25.570603000

6 7.208360000 -19.484120000 24.749081000

6 6.472511000 -18.204455000 24.495019000

1 7.921312000 -19.850475000 24.017561000

6 5.324069000 -18.439300000 23.531844000

1 7.136648000 -17.448357000 24.062895000

1 6.061314000 -17.808971000 25.434412000

7 5.291742000 -17.896803000 22.381943000

1 4.543475000 -19.131114000 23.871841000

6 4.203754000 -18.176180000 21.428938000

6 3.015759000 -18.978504000 21.918668000

1 4.673219000 -18.679194000 20.573345000

1 3.870103000 -17.200230000 21.059760000

6 2.959353000 -20.367793000 21.745553000

6 1.951939000 -18.339754000 22.572515000

6 1.866314000 -21.102831000 22.209422000

1 3.775929000 -20.876226000 21.237123000

6 0.857910000 -19.070229000 23.037667000

1 1.985494000 -17.260996000 22.710269000

1 1.836053000 -22.179151000 22.061392000

6 0.812530000 -20.454926000 22.856665000

1 0.038269000 -18.559251000 23.536309000

1 -0.040844000 -21.024536000 23.214916000

^2^**PC**_ring_

26 7.528831000 -14.483361000 21.690037000

7 5.534031000 -14.404857000 22.036615000

6 4.916259000 -14.275923000 23.254440000

6 3.486588000 -14.207132000 23.076189000

6 3.252748000 -14.270702000 21.735258000

6 4.539136000 -14.385492000 21.091835000

1 2.771590000 -14.099460000 23.882121000

1 2.306008000 -14.227190000 21.211579000

6 4.724654000 -14.444724000 19.717797000

1 3.839097000 -14.426751000 19.090458000

7 7.179167000 -14.538840000 19.706608000

6 5.956910000 -14.524241000 19.084193000

6 6.131820000 -14.639595000 17.657161000

6 7.470510000 -14.738796000 17.430672000

6 8.118605000 -14.679298000 18.717795000

1 5.322516000 -14.649291000 16.938159000

1 7.989752000 -14.845915000 16.486814000

6 9.490644000 -14.766547000 18.906238000

1 10.111097000 -14.875966000 18.022568000

7 9.514467000 -14.570789000 21.350311000

6 10.129467000 -14.711185000 20.135714000

6 11.562452000 -14.760651000 20.311711000

6 11.798788000 -14.635054000 21.645133000

6 10.510319000 -14.512379000 22.286990000

1 12.276772000 -14.870482000 19.505696000

1 12.748024000 -14.620914000 22.165500000

6 10.329093000 -14.343453000 23.651552000

1 11.216069000 -14.314471000 24.276326000

7 7.873856000 -14.231999000 23.662385000

6 9.098406000 -14.221335000 24.281529000

6 8.926123000 -14.096756000 25.708046000

6 7.585542000 -14.054359000 25.942427000

6 6.933599000 -14.150228000 24.659505000

1 9.738132000 -14.058444000 26.423026000

1 7.067784000 -13.971602000 26.889677000

6 5.557384000 -14.170496000 24.480717000

1 4.935699000 -14.079160000 25.365771000

8 7.543149000 -16.243822000 21.835502000

16 7.557145000 -12.171663000 21.388015000

1 7.717381000 -11.809281000 22.680383000

1 6.653199000 -16.680203000 21.933564000

1 7.009572000 -20.111266000 25.591693000

6 7.219613000 -19.493705000 24.724499000

6 6.483400000 -18.214466000 24.471706000

1 7.884659000 -19.891135000 23.964482000

6 5.328333000 -18.446814000 23.515981000

1 7.146041000 -17.460098000 24.032150000

1 6.080228000 -17.815147000 25.412537000

7 5.287741000 -17.901000000 22.367878000

1 4.549944000 -19.139203000 23.859849000

6 4.192086000 -18.177163000 21.422926000

6 3.006772000 -18.978985000 21.920152000

1 4.653980000 -18.679110000 20.562559000

1 3.856714000 -17.200102000 21.058254000

6 2.946386000 -20.367631000 21.743328000

6 1.949462000 -18.340310000 22.584572000

6 1.855749000 -21.102102000 22.213781000

1 3.757869000 -20.876051000 21.226777000

6 0.857908000 -19.070175000 23.056351000

1 1.986264000 -17.262067000 22.725495000

1 1.822333000 -22.177914000 22.062762000

6 0.808464000 -20.454244000 22.871503000

1 0.043305000 -18.559238000 23.563218000

1 -0.043020000 -21.023384000 23.234963000

^4^**TS**_reb._

26 7.021032000 -14.258408000 21.817179000

7 5.495825000 -13.036923000 22.348239000

6 5.313546000 -12.433227000 23.569682000

6 4.162367000 -11.566065000 23.521890000

6 3.668659000 -11.632912000 22.254545000

6 4.515467000 -12.541358000 21.521783000

1 3.800540000 -10.978854000 24.356369000

1 2.818020000 -11.111731000 21.834087000

6 4.372621000 -12.834140000 20.173391000

1 3.544294000 -12.372593000 19.645406000

7 6.311215000 -14.325376000 19.934798000

6 5.211488000 -13.667743000 19.444579000

6 5.023530000 -13.995142000 18.053067000

6 6.011847000 -14.869949000 17.716366000

6 6.807534000 -15.078855000 18.900485000

1 4.228271000 -13.603661000 17.431491000

1 6.195454000 -15.344559000 16.760825000

6 7.890651000 -15.944708000 18.971622000

1 8.166280000 -16.474327000 18.065163000

7 8.454672000 -15.601528000 21.333933000

6 8.649965000 -16.182394000 20.108906000

6 9.792468000 -17.064783000 20.157647000

6 10.289959000 -16.993685000 21.422494000

6 9.451253000 -16.068502000 22.148692000

1 10.157829000 -17.645297000 19.319959000

1 11.148856000 -17.504048000 21.839735000

6 9.654748000 -15.695179000 23.469969000

1 10.490283000 -16.146531000 23.995867000

7 7.784526000 -14.111772000 23.678869000

6 8.871331000 -14.789399000 24.173495000

6 9.070173000 -14.451592000 25.561060000

6 8.078798000 -13.581058000 25.902419000

6 7.271833000 -13.384372000 24.724158000

1 9.865829000 -14.844301000 26.181615000

1 7.892002000 -13.113775000 26.861019000

6 6.128467000 -12.595915000 24.680590000

1 5.853847000 -12.062833000 25.585340000

8 5.935976000 -15.746303000 22.337059000

16 8.364077000 -12.430510000 21.064456000

1 9.033851000 -12.206584000 22.217120000

1 5.091045000 -15.360412000 22.627573000

1 6.923482000 -17.311998000 25.678362000

6 5.960471000 -17.401795000 25.184280000

6 5.307611000 -18.736885000 25.098164000

1 5.327185000 -16.523285000 25.252867000

6 5.956070000 -18.105006000 23.840898000

1 4.224279000 -18.786730000 25.094750000

1 5.811680000 -19.610334000 25.502804000

7 5.066336000 -17.635941000 22.872263000

1 6.896361000 -18.554252000 23.512962000

6 5.225162000 -18.252504000 21.560073000

6 4.477136000 -19.579457000 21.534086000

1 6.282048000 -18.413029000 21.302711000

1 4.802568000 -17.565255000 20.822039000

6 5.164368000 -20.793367000 21.421847000

6 3.078436000 -19.601764000 21.635244000

6 4.470957000 -22.006074000 21.399220000

1 6.249172000 -20.788723000 21.343614000

6 2.383973000 -20.810622000 21.616760000

1 2.538277000 -18.663382000 21.731180000

1 5.019046000 -22.939962000 21.303804000

6 3.079051000 -22.017662000 21.497688000

1 1.299532000 -20.812399000 21.691891000

1 2.537824000 -22.959973000 21.480050000

^2^**TS**_reb._

26 7.236927000 -14.394258000 22.020168000

7 5.379546000 -13.621420000 22.346995000

6 4.911529000 -12.980515000 23.466794000

6 3.536566000 -12.580987000 23.277986000

6 3.180338000 -12.988639000 22.029195000

6 4.339334000 -13.633767000 21.455918000

1 2.939071000 -12.056757000 24.013069000

1 2.229585000 -12.867131000 21.525730000

6 4.383517000 -14.178686000 20.175957000

1 3.475625000 -14.116228000 19.583045000

7 6.714385000 -14.952994000 20.163323000

6 5.487580000 -14.772731000 19.576343000

6 5.491068000 -15.312492000 18.235581000

6 6.734634000 -15.821248000 18.021201000

6 7.488617000 -15.594415000 19.232498000

1 4.644497000 -15.293014000 17.560923000

1 7.122381000 -16.305517000 17.133888000

6 8.807225000 -15.986579000 19.429794000

1 9.307011000 -16.491838000 18.608566000

7 9.066816000 -15.147104000 21.712216000

6 9.538564000 -15.776714000 20.592976000

6 10.915344000 -16.175965000 20.780408000

6 11.270133000 -15.770478000 22.028828000

6 10.108788000 -15.126254000 22.601891000

1 11.515640000 -16.690161000 20.040522000

1 12.223074000 -15.883128000 22.530332000

6 10.077243000 -14.563328000 23.871135000

1 10.989086000 -14.621262000 24.458509000

7 7.760111000 -13.762571000 23.876208000

6 8.985036000 -13.935267000 24.462140000

6 8.988000000 -13.376337000 25.794585000

6 7.743983000 -12.866748000 26.007929000

6 6.984611000 -13.113664000 24.804512000

1 9.837999000 -13.383377000 26.465170000

1 7.360190000 -12.368476000 26.889184000

6 5.656407000 -12.744974000 24.617112000

1 5.161729000 -12.229935000 25.435332000

8 6.729035000 -15.999461000 22.784878000

16 7.803065000 -12.314067000 21.098405000

1 7.544682000 -11.531485000 22.170308000

1 6.146874000 -15.819604000 23.541231000

1 8.537296000 -19.252504000 23.461956000

6 7.542316000 -18.988922000 23.809171000

6 6.500400000 -20.039279000 23.879926000

1 7.500135000 -18.157599000 24.503447000

6 6.453294000 -18.979711000 22.738227000

1 5.716009000 -19.950054000 24.623464000

1 6.733702000 -21.052586000 23.564349000

7 5.464656000 -18.018296000 22.853022000

1 6.738619000 -19.336472000 21.745530000

6 4.675665000 -17.804178000 21.650877000

6 3.502226000 -18.778492000 21.654140000

1 5.264708000 -17.935463000 20.731410000

1 4.308041000 -16.774405000 21.670203000

6 3.421028000 -19.811875000 20.713842000

6 2.492166000 -18.661494000 22.619910000

6 2.346026000 -20.704234000 20.727007000

1 4.199672000 -19.913125000 19.961196000

6 1.420252000 -19.552836000 22.637411000

1 2.554061000 -17.867839000 23.359969000

1 2.292968000 -21.496665000 19.984790000

6 1.343523000 -20.577427000 21.689453000

1 0.642077000 -19.447808000 23.389118000

1 0.506766000 -21.270863000 21.701302000

^4^**PC**_reb._

26 7.454653000 -13.992963000 21.645869000

7 5.478699000 -14.101415000 22.110217000

6 4.929039000 -14.083203000 23.376995000

6 3.502486000 -13.919303000 23.301599000

6 3.185440000 -13.812422000 21.980588000

6 4.417317000 -13.913352000 21.245894000

1 2.842067000 -13.883080000 24.158530000

1 2.211116000 -13.672458000 21.530176000

6 4.517081000 -13.825393000 19.867199000

1 3.600664000 -13.685160000 19.303252000

7 6.956679000 -14.086617000 19.702809000

6 5.703152000 -13.918954000 19.155054000

6 5.784895000 -13.882259000 17.719556000

6 7.098702000 -14.037482000 17.395321000

6 7.820725000 -14.165956000 18.632647000

1 4.935999000 -13.762607000 17.058703000

1 7.551946000 -14.070651000 16.412926000

6 9.189980000 -14.363998000 18.715503000

1 9.748470000 -14.409202000 17.786163000

7 9.351357000 -14.480085000 21.161484000

6 9.894783000 -14.516244000 19.897934000

6 11.318758000 -14.723890000 19.968466000

6 11.639232000 -14.802268000 21.288499000

6 10.410470000 -14.644191000 22.024331000

1 11.972584000 -14.796407000 19.108841000

1 12.611411000 -14.952991000 21.740103000

6 10.324454000 -14.651277000 23.406820000

1 11.242450000 -14.787081000 23.969491000

7 7.891838000 -14.338428000 23.575138000

6 9.144749000 -14.516762000 24.122931000

6 9.059482000 -14.578502000 25.556645000

6 7.742159000 -14.447096000 25.880651000

6 7.021040000 -14.304270000 24.644779000

1 9.905838000 -14.716164000 26.217345000

1 7.284928000 -14.455810000 26.861755000

6 5.642292000 -14.179568000 24.560196000

1 5.081896000 -14.159087000 25.489300000

8 5.964508000 -17.108021000 21.259491000

16 7.699226000 -11.592230000 21.638542000

1 8.227085000 -11.515823000 22.881264000

1 5.762852000 -16.214199000 21.588649000

1 8.080652000 -18.545989000 24.044220000

6 7.029594000 -18.281590000 23.970245000

6 5.988185000 -19.351840000 24.184163000

1 6.789820000 -17.275043000 24.298306000

6 6.254618000 -18.816077000 22.798788000

1 5.055891000 -19.061662000 24.655920000

1 6.309770000 -20.366777000 24.401462000

7 5.259859000 -17.959886000 22.203050000

1 6.789013000 -19.466707000 22.099550000

6 4.279238000 -18.706742000 21.393857000

6 3.260820000 -19.434697000 22.247156000

1 4.780835000 -19.411428000 20.709131000

1 3.777101000 -17.958141000 20.772987000

6 3.094514000 -20.819766000 22.149911000

6 2.439590000 -18.718387000 23.130787000

6 2.129623000 -21.480980000 22.914933000

1 3.726529000 -21.386073000 21.469575000

6 1.479466000 -19.374685000 23.899446000

1 2.568484000 -17.642309000 23.215752000

1 2.014316000 -22.558185000 22.825753000

6 1.320475000 -20.760042000 23.792986000

1 0.850416000 -18.806589000 24.580367000

1 0.570143000 -21.271236000 24.390506000

^2^**PC**_reb._

26 7.001622000 -14.322518000 21.773299000

7 5.534751000 -13.051816000 22.342573000

6 5.406106000 -12.469603000 23.583378000

6 4.326622000 -11.513182000 23.575057000

6 3.810305000 -11.510787000 22.314671000

6 4.576973000 -12.461251000 21.548726000

1 4.017827000 -10.925746000 24.430590000

1 2.992332000 -10.919917000 21.922231000

6 4.391460000 -12.719337000 20.197433000

1 3.592859000 -12.187459000 19.689419000

7 6.226865000 -14.321672000 19.915212000

6 5.169821000 -13.585318000 19.441366000

6 4.983269000 -13.824746000 18.029225000

6 5.943841000 -14.711099000 17.653564000

6 6.714635000 -15.015840000 18.837278000

1 4.213745000 -13.364393000 17.422504000

1 6.127488000 -15.132855000 16.673421000

6 7.775666000 -15.910019000 18.866777000

1 8.050416000 -16.388118000 17.931415000

7 8.308184000 -15.757071000 21.261423000

6 8.509892000 -16.251317000 19.995018000

6 9.596065000 -17.201889000 19.997502000

6 10.057525000 -17.270318000 21.276587000

6 9.256123000 -16.358665000 22.055838000

1 9.947958000 -17.736386000 19.124079000

1 10.867410000 -17.870995000 21.671057000

6 9.438606000 -16.111100000 23.410182000

1 10.230628000 -16.652692000 23.918524000

7 7.668029000 -14.434958000 23.668585000

6 8.697454000 -15.203117000 24.155450000

6 8.909931000 -14.929366000 25.556343000

6 8.001048000 -13.979471000 25.910609000

6 7.230329000 -13.677466000 24.727463000

1 9.663303000 -15.405912000 26.170979000

1 7.851760000 -13.515944000 26.877721000

6 6.187244000 -12.760867000 24.693235000

1 5.962598000 -12.229082000 25.612889000

8 5.300030000 -15.833658000 22.207091000

16 8.396770000 -12.653355000 21.255816000

1 9.502238000 -13.136534000 21.867448000

1 4.625376000 -15.272557000 22.623888000

1 6.160759000 -16.659453000 25.808053000

6 5.387604000 -16.998925000 25.125618000

6 5.275299000 -18.465498000 24.802276000

1 4.465038000 -16.425988000 25.163083000

6 5.826385000 -17.503868000 23.776814000

1 4.284976000 -18.868965000 24.622281000

1 5.973677000 -19.162704000 25.256516000

7 4.985324000 -17.191629000 22.648189000

1 6.898625000 -17.534987000 23.576379000

6 5.249339000 -18.032924000 21.465467000

6 4.711621000 -19.437694000 21.643449000

1 6.324005000 -18.061532000 21.229669000

1 4.745247000 -17.537786000 20.630184000

6 5.566268000 -20.543650000 21.593021000

6 3.338471000 -19.654136000 21.829663000

6 5.064506000 -21.841374000 21.724233000

1 6.632854000 -20.387086000 21.448671000

6 2.834780000 -20.946839000 21.966501000

1 2.668368000 -18.799419000 21.874036000

1 5.742693000 -22.689701000 21.681130000

6 3.697671000 -22.045895000 21.913059000

1 1.768118000 -21.099333000 22.110021000

1 3.304722000 -23.053918000 22.016326000
